# Multidimensional Evolution of Carbon Structures Underpinned by Temperature‐Induced Intermediate of Chloride for Sodium‐Ion Batteries

**DOI:** 10.1002/advs.201800080

**Published:** 2018-03-25

**Authors:** Peng Ge, Hongshuai Hou, Xiaoyu Cao, Sijie Li, Ganggang Zhao, Tianxiao Guo, Chao Wang, Xiaobo Ji

**Affiliations:** ^1^ State Key Laboratory of Powder Metallurgy College of Chemistry and Chemical Engineering Central South University Changsha 410083 China; ^2^ College of Chemistry Chemical and Environmental Engineering Henan University of Technology Zhengzhou 450000 China; ^3^ School of Energy Science and Engineering University of Electronic Science and Technology of China Chengdu 611731 China

**Keywords:** carbon anodes, chloride, electrochemistry, sodium‐ion batteries

## Abstract

Different dimensions of carbon materials with various features have captured numerous interests due to their applications on the tremendous fields. Restricted by the raw materials and devices, the controlling of their morphology is a major challenge. Utilizing the catalytic features of the intermediates from the low‐cost salts and polymerization of 0D carbon quantum dots (CQDs), 0D CQDs are expected to self‐assemble into 1/2/3D carbon structures with the assistance of temperature‐induced intermediates (e.g., ZnO, Ni, and Cu) from the salts (ZnCl_2_, NiCl_2_, and CuCl). The formation mechanisms are illustrated as follows: 1) the “orient induction” to evoke “vine style” growth mechanism of ZnO; 2) the “dissolution–precipitation” of Ni; and 3) the “surface adsorption self‐limited” of Cu. Subsequently, the degree of graphitization, interlayer distance, and special surface area are investigated in detail. 1D structure from 700 °C as anode displays a high Na‐storage capacity of 301.2 mAh g^−1^ at 0.1 A g^−1^ after 200 cycles and 107 mAh g^−1^ at 5.0 A g^−1^ after 5000 cycles. Quantitative kinetics analysis confirms the fundamentals of the enhanced rate capacity and the potential region of Na‐insertion/extraction. This elaborate work opens up an avenue toward the design of carbon with multidimensions and in‐depth understanding of their sodium‐storage features.

## 1 Introduction

Carbon, as most closely related to human, has been distributed spaciously in the nature, and then spontaneously devoted to enormous attentions in physical and chemical properties. Interestingly, various atomic hybrid orbitals were observed, including sp, sp^2^, and sp^3^ characteristics.^1^ Moreover, the anisotropy of crystal and other arrangements are dominated by that of sp^2^, giving rise to the innumerable characteristics. In fact, carbon is unique among total elements as the single unit to form 0D fullerenes,^2^ 1D carbin and nanotube,^3^ 2D graphene sheets and 3D diamond crystal,^4^ which are utilized in a myriad of applications, such as biosensors, catalyst, renewable energy storage, and so on.[[qv: 4b]] In addition, allotropes of carbon have various properties, from hard (diamond) to soft (graphite), from insulative (diamond) to semiconductive (graphite) and conductive (graphene), and from light absorbing (graphite) to diaphanous (diamond).^5^ Considering its fascinating potential, numerous efforts were further made for carbon to explore different dimensions, and great progress was achieved as expected.^6^

Inspiringly, 0D carbon quantum dots (CQDs) are found,^7^ enclosing (1) the graphite CQDs came from the top‐down manner based on the target materials, containing carbon fiber, oxidized graphene, and carbonated organic products resulted from high temperature, which exhibit the lamellar structure and good crystallinity;[[qv: 7b,8]] (2) the polymerized CQDs derived from small organic molecules through the down‐top process (e.g., carbohydrate, citric acid, and amino acid), with abundant functional groups and weak crystallinity.^9^ Note that extreme conditions or special devices are necessary in most of the fabrication methods, seriously restraining the advancements of CQDs. Recently, facile and effective organic CQDs are prepared with extensive organic functional groups, which have been successfully applied in sodium‐ion batteries (SIBs) by transferring themselves to 3D structures.[[qv: 9a,b]] With this evolution, the carbon materials would have large changes in their properties. For 1D carbon structures, it can be summarized to be of two main kinds: (1) carbon nanotubes (CNTs) mainly with sp‐hybridized carbon atoms, containing single‐walled and multiwalled carbon nanotubes, are deemed with wrapping graphite layers and perfect tubes in structure, showing the high modulus, strength, and conductivity;^10^ (2) carbon fibers (CNFs) mostly with sp^2^‐hybridized carbon structure, displaying the nonsequence monofilaments with the length/diameter ratios larger than 100 times.^11^ As well known, graphene is the hot topic among 2D carbon structures, including graphene oxides (GOs) and reduced graphene oxides, which are composed of the linking of carbon's six‐atom rings to form a 2D honeycomb network.^12^ Surprisingly, treated with high energy consumption, graphene can be employed to buckle the spherical structures (0D fullerenes), 1D CNTs, and layered structures (3D graphite). The state‐of‐the‐art graphene has also been devoted to extensive attentions with its great physicochemical properties, such as outstanding electrical, thermal conductivity, and high specific surface area.^13^ As the highest dimensional carbon nanostructures, 3D structure is comprised of 0D CQDs to form bulk carbon, 1D CNFs to present the network, 2D graphene to fabricate microporous structures, which would effectively prevent the fundamental units from agglomerating, showing the stable frame structure.^14^

Carbon structures with various dimensions are obtained traditionally through unique strategies, which are seriously limited by raw materials and particular equipment. For instances, the electrospinning process was applied to fabricate 1D polymer nanofibers, which could be further carbonized to produce CNFs.^15^ Micromechanical exfoliation as the primitive approach is still grateful to effectively prepare 2D graphene at present.^16^ Although the catalytic chemical vapor deposition can be used to fabricate 1D and 2D carbon materials, the required devices and catalytic nanoparticles are essential, where iron is used to constitute the CNFs and copper is employed to assemble 2D graphene.^17^ Certainly, the whole dimensions of carbon nanostructures can also be obtained by the biomasses,^18^ but the raw materials are restricted. The bee pollen, cellulose, corn, and peat mosses are transformed into 0D CQDs, 1D CNFs, 2D graphene, as well as 3D macroscopic carbon frameworks, respectively. To date, controlling carbon morphologies is still unsolved. As motivated by the Antonietti and co‐workers' report,^19^ salts play an important role in the carbon target through the function of molten salt, which is ascribed that the salts would display numerous chemical and physical transformations with the raised temperature. Meanwhile, compared with the aforementioned manners, the controllable salt methods are experienced a renaissance with their favorable features, showing that it will be expected for an ideal technique to diverse carbon nanostructures more effectively.

In this study, for the first time, the CQDs are successfully evolved into 1D‐wrapped CNF, 2D large‐scale carbon nanosheets (CNSs), and 3D porous carbon framework (CFW). The corresponding formation mechanisms of those samples are investigated in details, showing that (1) the “orient induction” of ZnO, (2) the “dissolution–precipitation” of Ni, and (3) the “surface adsorption self‐limited” of Cu. For 1D structures, the vines' style growth mechanism was triggered. Such evolutions lead to varied electrochemical performances of the formed carbon structures. Most greatly, 1D CNFs display high reversible capacities of 301.2 mAh g^−1^ after 200 cycles at 0.1 A g^−1^ and 107 mAh g^−1^ after 5000 cycles at 5.0 A g^−1^.

## 2 Results and Discussion

### 2.1 The Evolution form 0D CQDs to 1/2/3D Carbon Structure

**Figure 1:**
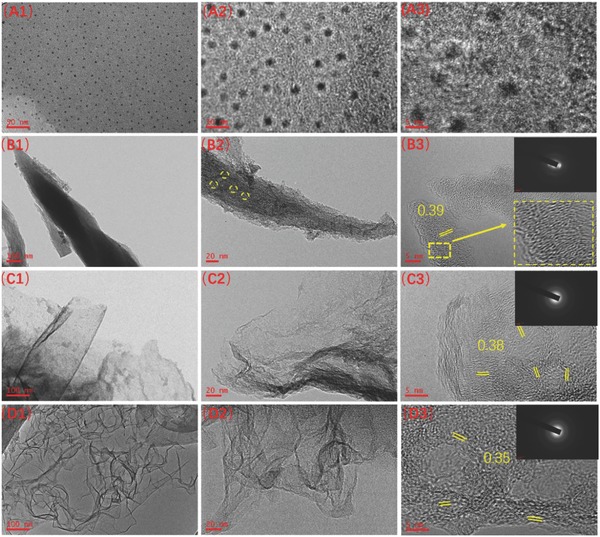
TEM and HRTEM images of A1–A3) 0D CQDs, B1–B3) 1D CNF, C1–C3) 2D CNS, and D1–D3) 3D CFW.

**Figure 2:**
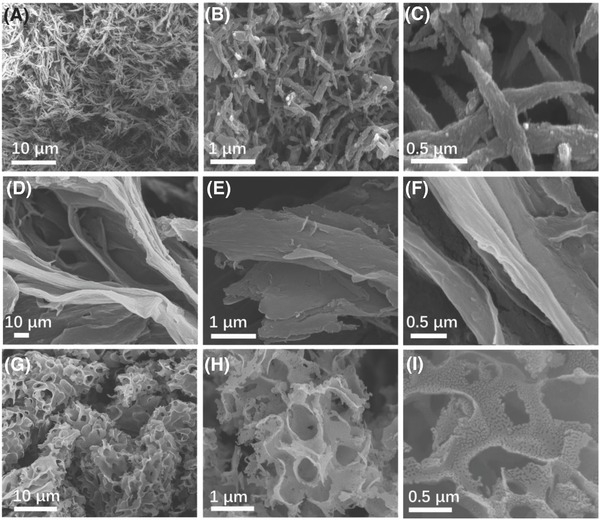
SEM images for A–C) 1D CNF, D–F) 2D CNS, G–I) and 3D CFW.

The effects of different salts on the microstructure of the as‐prepared samples were systematically investigated. The detailed morphologies and crystal structures of 1D CNF, 2D CNS, and 3D CFW were revealed by transmission electron microscopy (TEM) in **Figure** **1**. The images of 0D CQDs with different magnification are shown in Figure 1A, which were quite uniform and monodispersed with the diameters in the range of 3.0–5.0 nm. Without the introduction of any salts, 0D CQDs were instantly transformed into bulk carbon particles at 700 °C as shown in Figure 1S (Supporting Information). Impacted by ZnCl_2_, NiCl_2_, and CuCl, 0D CQDs were manipulated to 1D CNF, 2D CNS, and 3D CFW, respectively. Note that 1D CNF was formed through the wrapping of the small CNFs in Figure 1B, differing from that of the previous report.^11^ Moreover, the large‐scale sheet of 2D CNS is found, and the porous framework of 3D CFW is presented in Figure 1C,D. These interesting evolutions demonstrate that multidimensional carbon structures are surely shaped through different features of salts, which is worthy to explore their corresponding formation mechanisms. With the careful observation, it is found that numerous defects existed on the surface of CNFs display shuttle shapes, which were wrapped from small primary CNFs with the diameter of ≈80 nm in Figure 1B. Clearly, 2D‐inflected sheets are composed of some thin sheets, and there are plenty of microspores on the surface of 3D CFW structures in Figure 1C,D. As shown in the high‐resolution TEM (HRTEM) images of Figure 1B–D, the graphitic lattice spacing of 1D CNF, 2D CNS, and 3D CFW is ≈0.39 nm, ≈0.38 nm, ≈0.35 nm, respectively. Significantly, all of them would be beneficial for metal ions (e.g., Li^+^ and Na^+^) insertion/desertion, resulted from the relatively larger graphite lattice than that of graphite (0.335 nm).^20^ Moreover, the number of layer‐stacked‐graphene sheets decreases in sequence: 1D CNF > 2D CNS > 3D CFW, which is further confirmed through the following X‐ray diffraction (XRD) analysis in **Figure** **2**. The reason of the observed diverse lattice distances is deduced that the van der Waals' forces of tiny sheets in the microcrystalline graphite can be altered by the salts in the carbonization process.^21^ In addition, note that the brightness of the selected area electron diffraction (SAED) images is improved in the order of 1D CNF < 2D CNS < 3D CFW, showing the increased degree of graphite. Interestingly, the elliptical diffraction ring and long‐term disordered lattice of 1D CNF are noticed, demonstrating that the preferred orientation is displayed by the growth of carbon atoms.

**Figure 3:**
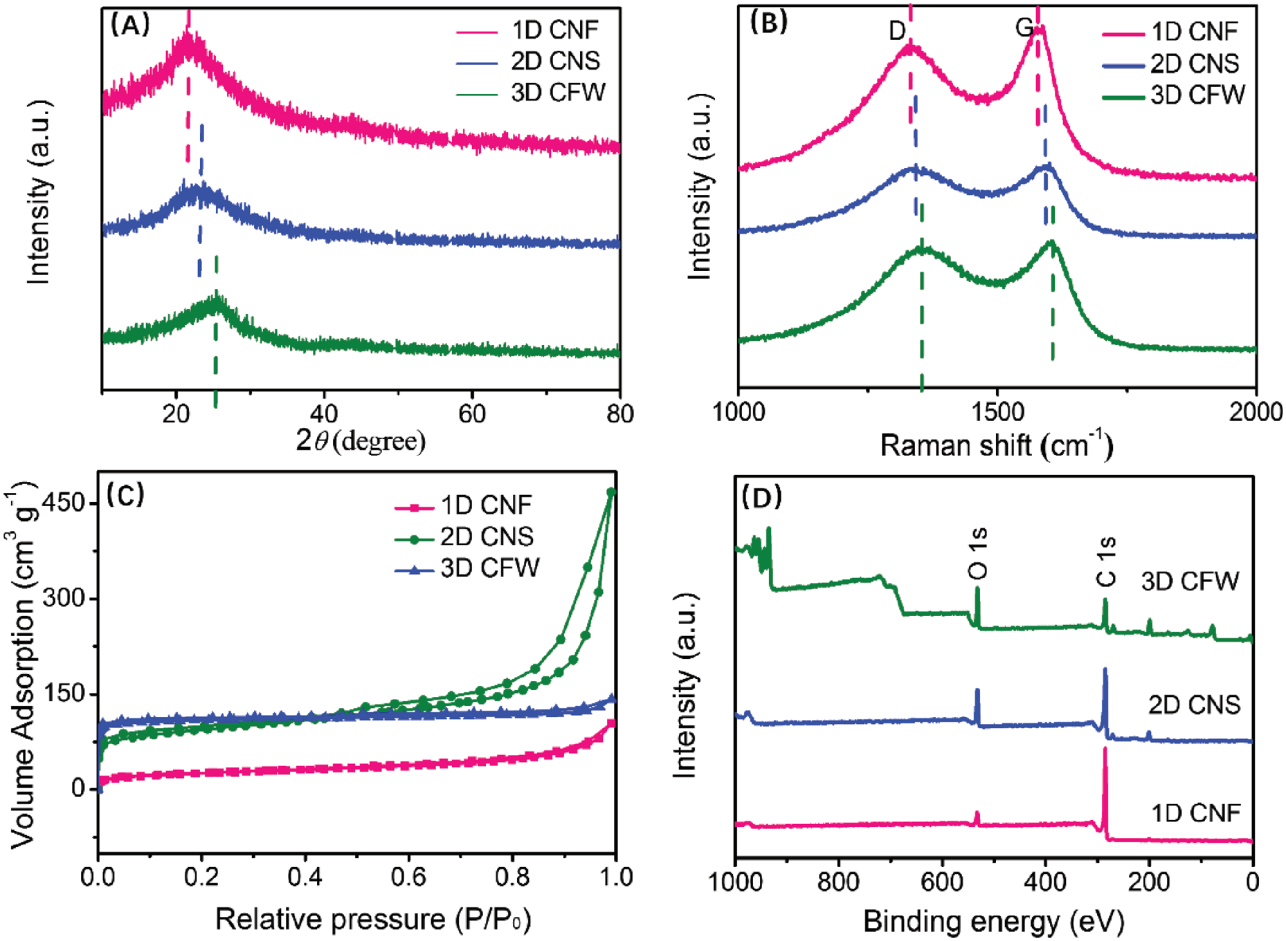
A) X‐ray diffraction (XRD) patterns, B) Raman spectroscopy, C) nitrogen adsorption–desorption isotherm, and D) X‐ray photoelectron spectroscopy (XPS) of 1D CNF, 2D CNS, and 3D CFW.

The large‐scale morphology of the as‐obtained samples was also investigated through scanning electron microscopy (SEM). Without salts, CQDs are carbonized to irregular bulk carbon in Figure 1S (Supporting Information). The uniformly distributed CNFs with the length of 2–8 µm are obtained as shown in **Figure** **3**A, suggesting that the ZnCl_2_ molten salt (melting point, 290 °C) provides a stable reaction matrix and the in situ formed ZnO brings adequate active sites to favor the formation of 1D structure. As depicted in Figure 3B,C, a mean diameter of shuttle shape CNF with rough surface is about 150 nm, indicating that numerous defects were existed in CNF, which can facilitate the insertion–extraction of sodium ions. Meanwhile, owing to the catalysis functions of the Ni plate from the NiCl_2_, the large‐area CNS is observed in Figure 3D, presenting the bending and uneven characteristics. In Figure 3E, the carbon sheets consist of 3–5 monolayer carbon papers with harsh surface. When 0D CQDs were assembled into 3D CNF, the tremendous framework was produced with bubbles' structure as shown in Figure 3G. To examine the surface carefully, many pores with diameters of 200 nm are found, in Figure 3G–I, coming from the removal of large Cu particles formed by the reduction of CuCl with carbon. The corresponding mechanisms were further vitrified through the intermediate productions in Figure 2S (Supporting Information). It is proved that the catalysts of multidimensional carbon structure are ZnO, Ni, and Cu, leading to the evolution of internal structures. Compared to the morphology of salts in Figure 3S (Supporting Information), it is noteworthy that the self‐generated intermediate products (ZnO, Ni, and Cu) are crucial for the self‐assembly of carbon atoms to 1/2/3D structures.

Obviously, with the introduction of carbon sources at the high temperature (700 °C), the chemical and physical properties of salts would have an enormous transformation: (1) ZnCl_2_ was turned into fluid accompanied with ZnO to supply stable matrix; and (2) NiCl_2_ and CuCl were chemically reduced to metal substances by the intermediately produced carbon as demonstrated in Figure 2S (Supporting Information). It is clear that the crystallographic structures of the carbon target were regulated by diverse “calcination partners,” which were clarified by XRD as shown in Figure 2A. Broad (002) and weak (100) diffraction peaks located at ≈23^o^ and ≈44^o^ are observed, which are classical for the turbostratic structure from the pyrolysis of high polymers due to the crosslinking features of precursors and their unique chemical structure.^22^ Note that the crystalline nature of 1D CNF was better as compared to 2D CNS and 3D CFW, which was deduced to the following reasons: (1) considering heat conductivity coefficient, that of liquid is always higher than that of gas, suggesting that ZnCl_2_ molten salt plays significant roles in favoring the heat transfer; (2) benefitting from ZnCl_2_ as a dehydrator and the abundant oxygen of CQDs, the nascent oxygen triggers a cascading effect, contributing to the disentanglement arrangement of carbon atoms, which agrees well with the previous reports.^23^ Moreover, the shifting of (002) peaks toward lower angle is observed in the order of 1D CNF (22.3°) < 2D CNS (23.1°) < 3D CFW (25.2°). Based on the Bragg's equation, the lattice spaces (*d*_002_) are estimated to be 1D CNF (0.397 nm) > 2D CNS (0.385 nm) > 3D CFW (0.356 nm), all of which are larger than that of the model carbon (i.e., graphite, 0.335 nm), favoring the insertion of metallic ions (e.g., Li^+^ and Na^+^) in the context.^24^ Certainly, there is some microcrystalline graphite in the disordered carbon, formed by the layer‐stacked‐graphene sheets. Utilizing the scatter' equation and the full width at half‐maximum of (002) from the peaks finding reports, the *c*‐axis length (*L_c_*) can be calculated for 1D CNF (1.45 nm), 2D CNS (0.792 nm), 3D CFW (0.398 nm), representing that the as‐obtained carbonized samples are consisted of 5–6 sheets (2.35/0.397 = 5.9), 4–5 sheets (1.88/0.385 = 4.8), and 4–5 sheets (1.56/0.356 = 5.3), as discussed in **Table** **1**. For 2D CNS, the analysis of atomic force microscopy (AFM) displayed the thickness about 0.178 nm (Figure 17S, Supporting Information). More layer‐stacked‐graphene sheets are concluded to this reason: the abundant aldehyde (—CHO) and carbonyl (—C=O) groups existed CQDs coming from acetone, and the molten ZnCl_2_ as the coupling reagent facilitate the aldol reaction on the surface of active sites, which are faster than the formation of 2D CNS and 3D CFW, giving rise to more generated carbon sheets. Thus, the oxygen‐functional groups of the as‐derived samples were explored as shown in Figure 2D and Figure 5S (Supporting Information). Obviously, the oxygen contents of the samples are increased in order of 1D CNF (7.37%) < 2D CNS (19.2%) < 3D CFW (33.6%), revealing the catalytic advantages of ZnCl_2_ in the aldol reaction. From the Fourier transform infrared spectroscopy (FTIR) analysis of Figure 5S (Supporting Information), it is found that the residual groups of 0D CQDS are mainly C—O—C, C—OH, and C=O.

**Table 1:** Structure parameters of the as‐derived samples

		Θ [^o^]	*d*_(002)_ [nm]	*L_c_*	*L_c_*/*d*_(002)_	*I*_D_/*I*_G_	BET [m^2^ g^−1^]
1D	CNF‐400	23.5	0.373	1.89	4.9	1.43	697.3
	CNF‐500	23.2	0.378	1.96	5.1	1.27	841.2
	CNF‐600	22.7	0.386	2.0	5.4	1.23	687.5
	CNF‐700	22.3	0.397	2.35	5.9	1.15	173.3
2D CNS		23.1	0.385	1.88	4.8	0.83	338.4
3D CFW		25.3	0.356	1.56	4.3	0.58	429.5

In order to further explore the effect of salts on the construction and extent of disordered and graphitic structure of the carbonized samples, Raman spectroscopy was conducted as displayed in Figure 2B. Two separate characteristic peaks of the as‐derived samples are exhibited, where D is related to the disordered lattice or defects in the conjugated π system (sp^3^‐type carbon), and G is associated with the crystalline graphite (sp^2^‐type carbon).[[qv: 14a,25]] For 1D CNF, the half width at half maximum of G and D bands is larger than those of 2D CNS and 3D CFW, and the right shift of peaks is also observed, suggesting the less content of ordered hexagonal structures.^26^ Note that Ni or Cu has a catalytic action in favoring the better graphene structure.^27^ From the low fitted *A*_sp3_/*A*_sp2_ value (the integrated area of sp^3^ to sp^2^) in Figure 5S (Supporting Information), the relative ratio of *I*_D_/*I*_G_ is about 1.15, 0.83, or 0.58 in Table 1S (Supporting Information), respectively. Greatly, the enlarged graphitized carbon in the order of 1D CNF < 2D CNS < 3D CFW is found, which can be attributed to the fact that the graphitic of lattice for 1D CNF possesses more defects as compared to those of other samples, thus enhancing the Na‐storage properties.^28^ Considering the result of *L_c_*,^22^ nanosized voids in the hard carbons are deduced to be created in the spaces of graphite microstructure. The specific surface area and pore distribution were also determined, displaying 173.3 m^2^ g^−1^ for 1D CNF, 338.4 m^2^ g^−1^ for 2D CNS, and 429.5 m^2^ g^−1^ for 3D CFW, which are reasonable for different dimensional structures, and 3D structure supplies the largest space as compared to other samples. In addition, 1D CNF possesses the biggest microspores with a mean diameter of 3.43 nm as well as the largest pores volume in Figure 5S (Supporting Information), which is well consistent with the analysis of Raman, resulted from the activation of ZnCl_2_.^29^

**Scheme 1:**
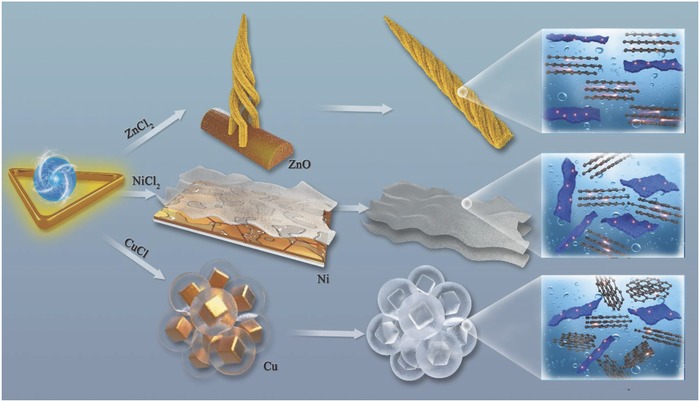
Schematic illustration of 1D CNF, 2D CNS, and 3D CFW.

**Figure 4:**
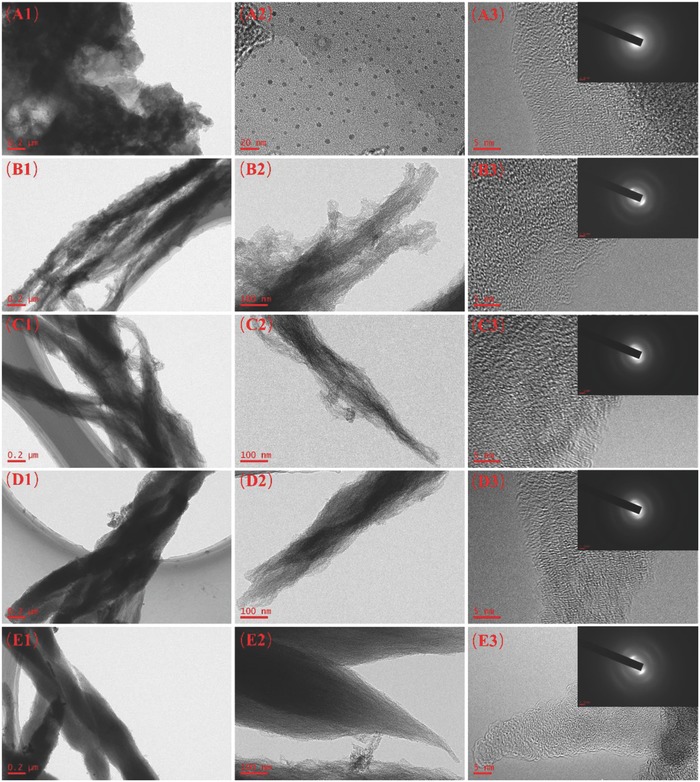
TEM and HRTEM images, SAED patterns of A) CNF‐300, B) CNF‐400, C) CNF‐500, D) CNF‐600, and E) CNF‐700.

**Scheme 2:**
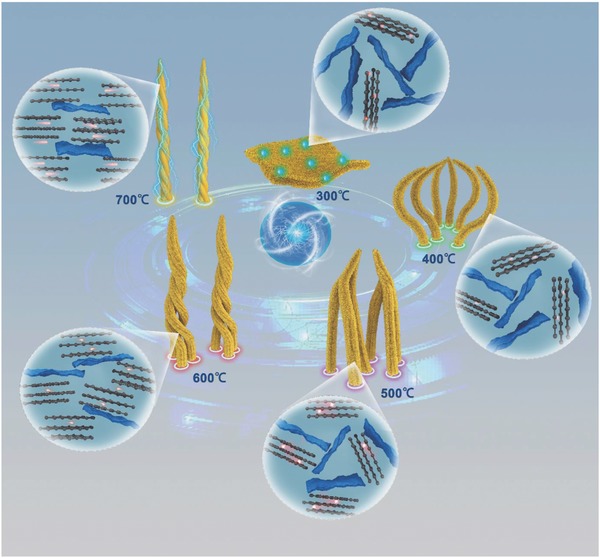
Schematic illustration of 1D CNF in detail.

**Figure 5:**
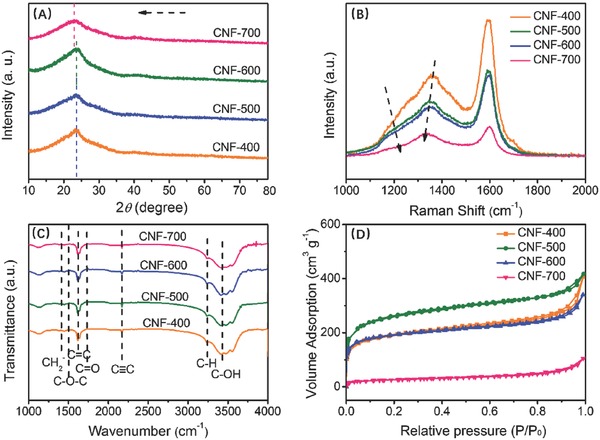
A) XRD, B) Raman, C) FTIR, and D) specific surface area (Brunauer–Emmett–Teller; BET) of CNF‐400/500/600/700.

Stimulated by this interesting evolution of microstructure, the corresponding mechanisms are proposed for the effect of salts on morphology in **Scheme** **1**. As shown by the previous report,^30^ numerous align active sites existed on the surface of ZnO, which could promote the development of carbon atoms. Through the transformation of ZnCl_2_ to ZnO above 400 °C and the plentiful functional groups (C=O, C—OH) of 0D CQDs, as confirmed in Figure 5 and Figure 5S (Supporting Information), 1D CNF was expected to be formed. It is clear that the features of 0D CQDs with good dispersibility are stable as displayed in **Figure** **4** with a low temperature (300 °C). When the mixture of 0D CQDs and ZnCl_2_ was bought above 400 °C, 0D CQDs were pyrolyzed to carbon atoms, which were assembled into rods, further being wrapped into 1D shuttle structure. The more detailed mechanism is exhibited in **Scheme** **2**. Notably, the replacement of NiCl_2_ has enormously significant effects on the microstructure to transform 0D CQDs to 2D CNS. Due to the presence of carbon atoms and the high melting point of NiCl_2_ (1001 °C), bulk Ni particles with large flat surface were produced through the reduction of NiCl_2_, which is ascribed to NiCl_2_ with flat morphology as a template (Figure 3S (Supporting Information)). Interestingly, the carbon atoms were dissolved in the Ni atoms at high temperature, which could result in the precipitation of carbon atoms on the surface of Ni materials, finally forming the large‐scale nanosheets.^31^ However, the multilayer sheets were shown in Figure 3, resulted from the uncontrollable velocity of the extraction for carbon atoms. In comparison with the large NiCl_2_ particles, the small particles of CuCl were displayed in Figure 3S (Supporting Information), which could be wrapped by the colloid CQDs. In addition, the cubic Cu with the diameter of 500 nm in Figure 2S (Supporting Information) was reduced from the CuCl, derived from the unique characteristic of CuCl, which is used as a catalyst and a pore‐foaming agent. The carbon film was formed around the Cu templates, further connecting to the porous framework. Moreover, the solubility of carbon atoms in Cu is much smaller than that of Ni;^31^ its growth mechanism was deemed as surface adsorption self‐limited, that is, the catalytic ability of Cu was shielded by the formed structure, which could facilitate the formation of the thin carbon film. With the removal of Cu, the big pores were finally produced as shown in Figure 3. Note that the catalysts in the reaction are unstable, and it is due to the fact that the differences in temperature play an important role in obtaining the target materials. With the rapid increase of temperature, ZnO, Ni, and Cu are formed gradually, and some CQDS were attached to their surface. When the temperature reached 700 °C, the catalysts with activity have been obtained, which can effectively induce the evolution of 0D CQDs.^32^

### 2.2 The Growth of 1D CNF‐400/500/600/700s

Owing to the fascinated transformation from 0D CQDs to 1D CNF, it is interesting to explore the effect of calcination temperatures (300, 400, 500, 600, and 700 °C) on its evolution process. The formation mechanism and microstructure were investigated through TEM, HRTEM, and SAED patterns, as shown in Figure 4. Obviously, 0D CQDs were not pyrolyzed to carbon atoms, which were still dispersed uniformly in ethanol in Figure 4A2. Interestingly, with the increased temperature, the higher catalytic activity was aroused by more thermal energy, leading to the fact that the shuttle structure of CNF is composed of thin primitive rods, which were wrapped more tightly in Figure 4B1–E1. The growth mechanism of CNF is similar to that of rattans in nature, which all depends on the strong interaction force.^33^ The mechanism was further demonstrated by the gradually closing cusps in the magnification TEM of the cusps in Figure 4B2–E2. Moreover, no apparent long‐range ordered structure is found in HRTEM images of CNFs, but some obvious turbostratic graphitic microstructures are observed, revealing the amorphous nature of the samples of hard carbon materials.^34^ With the increased carbonization temperatures, different ranges of structures constructed by the hexagonal layer of carbon and the layer‐stacked‐graphene sheets grow evidently, which is attributed to the fact that the orientated growth of carbon atoms was induced by ZnO. The increased disordered structures could supply more approaches for metal ions' insertion and plentiful surface defects, improving the adsorption ability.^35^ In Figure 8S (Supporting Information), the broken root is clearly observed, and the disordered lattice shows the growth direction. This type of preferred orientation could also be formed in SAED images with the rising long axis of ellipse, which are indexing to the characteristic peaks of carbon (002), agreed well with the analysis of XRD. The sharpness of ellipse is not uniform in Figure 4, perhaps resulted from the defects and lattice distortion. Moreover, no crystal diffraction spots appearing is another demonstration of amorphous carbon, and the improved degree of graphitization is further verified through the enhancing sharpness of diffraction rings.^36^ According to the equation (*λL* = *Rd*), the similar *d* value was calculated with the assistance of cycle radius from SAED patterns, which is consistence with the TEM analysis.

**Figure 6:**
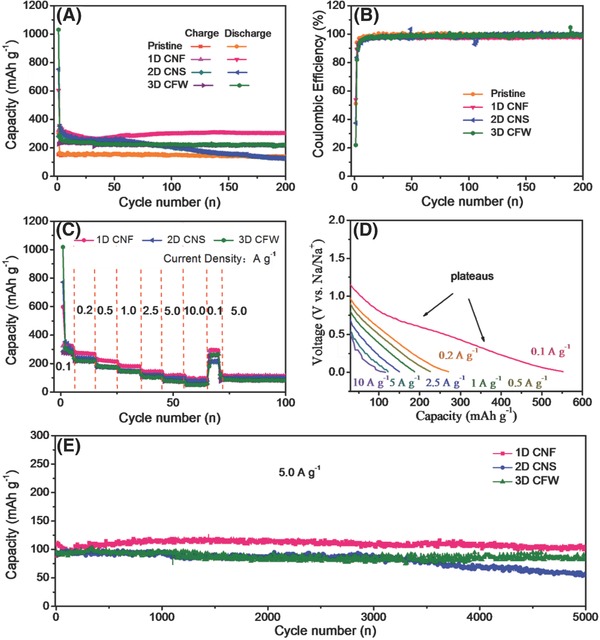
A) The cycling performances, B) Coulombic efficiency, C,E) and rate properties of 0D CQDs, 1D CNF, 2D CNS, and 3D CFW. D) The discharge platforms of 1D CNF.

**Figure 7:**
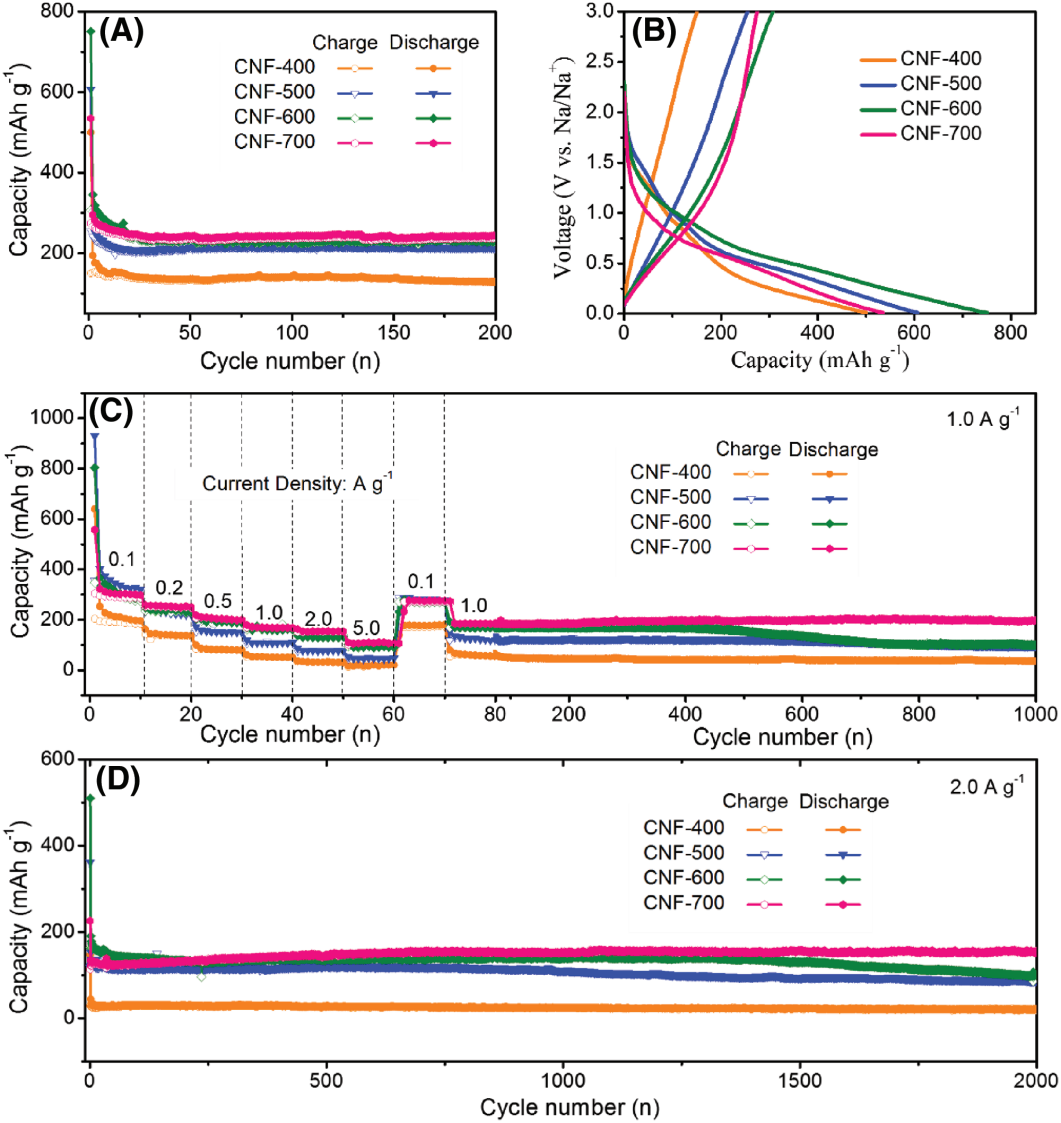
A) The cycling performances, B) Coulombic efficiency, C,E) and rate properties of 0D CQDs, 1D CNF, 2D CNS, and 3D CFW, and D) the discharge platforms of 1D CNF.

The SEM images of CNF‐400, CNF‐500, CNF‐600, and CNF‐700 were displayed in Figure 6S (Supporting Information). Figure 4S (Supporting Information) also presents the SEM images of CNF‐300 without any rod‐like structures, confirming that CNFs were not formed at low temperature. When the temperature increased to 400 °C, some ateliosis wires were wrapped into the shape of rods in Figure 6Sa,b (Supporting Information). Clearly, more formation energy was supplied at higher temperature to build the well‐shuttle shape but with some branches at the cusp of the sample in Figure 6Sc,d (Supporting Information). From the images of CNF‐600/700 in Figure 6Se–h (Supporting Information), the shuttle rods with a closed sharp corner were constructed completely and CNF‐600 displays intact rod structure as compared to CNF‐700, suggesting that CNF‐700 was broken in the rapid formation process with highest energy. Moreover, in Figure 7S (Supporting Information), the available ratio of ZnCl_2_ and the annealing times are vital for the formation of rod structure.

The detailed lattice spaces, degree of graphite, functional groups, and special surface area for 1D structures were investigated in **Figure** **5** and the corresponding fitting curves are shown in Figure 9S (Supporting Information). With improved carbonization temperature, the (002) peaks' positions were shifted toward a low angel (from 23.5^o^ to 22.3^o^), which are calculated as the lattice expands gradually from 0.373 to 0.397 nm, facilitating the insertion–extraction of Na^+^.^37^ In Table 1, the value of *L_c_*/*d*_(002)_ is found from 4.9 to 5.9, indicating the increased interlayer in the graphitic domain,^22^ which agrees well with the analysis of TEM in Figure 4. The Raman spectra of CNF‐400/500/600/700 were displayed in Figure 5B, showing the improved degree of graphite form the variation of *I*_D_/*I*_G_, which is beneficial for the shuttling of sodium ions at high‐rate current densities.^38^ Moreover, differing from the obtained carbon materials at high temperature, the peak at 1200 cm^−1^ (C—O) was found decreasing, suggesting the reduction of numerous oxygen‐containing groups.^39^ Plentiful functional groups were further confirmed in **Figure** **6**C, which are —CH_2_, —CH_3_, O—C—O, C=O, and C—OH, respectively. The special surface areas of the CNF‐400/500/600/700 samples are 697.3, 841.2, 687.5, and 173.3 m^2^ g^−1^, derived from the structure growth speeds for the carbonized temperature, which effectively demonstrated that the rods were intertwined more tightly.

The growth mechanism of 1D CNF is proposed, in detail, in Scheme 2. Note that 0D CQDs were not fully decomposed into the carbon and oxygen atoms, but rather agglomerated large organic particles at 300 °C. When the annealing temperature (AT) was increased to 400 °C, the formation of ZnO can successfully induce the formation of 1D structure. However, the weak catalytic activity prompts the generation of 1D CNF with very rough surface and insufficient energy, where the coiling cannot be triggered. With the improvement of AT to 500 °C, the activity of ZnO was further enhanced, facilitating carbon atoms gathering into the 1D structure, forming relatively smooth 1D CNF. Meanwhile, taking account of the unstable roots and more energy, the rods play an important role as the mutual support to maintain this grows upward, so the tips of rods have been wrapped to some extent. At high temperature (600 °C), the increased induction force accelerates the moving of carbon atoms toward the center of construction, leading to the reducing of scraps. The twine draws to an end, approximately forming the basiconic carbon structures. Eventually, the temperature (700 °C) makes the carbon atoms run up to the surface of 1D structure, which brings the relatively smooth rods with numerous defects. Note that the catalysts obtained from different temperatures have various activities.^40^ The wrapped process was accomplished to obtain the vine structure. In addition, the orientation of the crystalline lattice is boosted with the increased AT. In the nature, it is found that the formation mechanism of 1D structure is similar to that of vines,^33^ and all of them depend on the external force (magnetic force and induced force) and mutual supporting.

### 2.3 Electrochemical Properties

**Figure 8:**
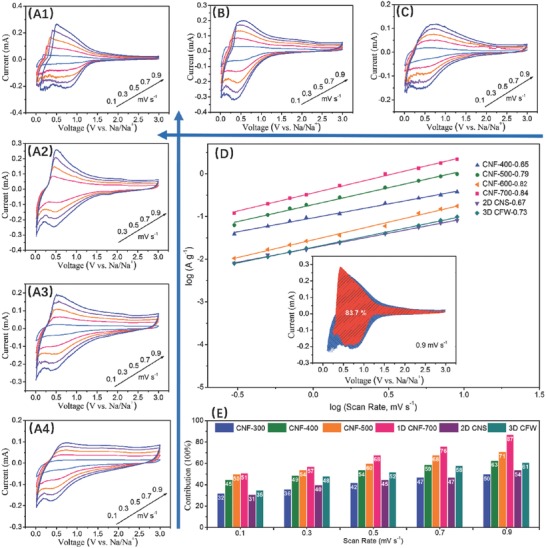
CV curves at various scan rates, capacitive contribution of the total current (orange) for A1–A4) 1D CNF700/600/500/400, B) 2D CNS, C) 3D CFW, D) log *i* versus log *v* plots and separation of the capacitive and diffusion‐controlled charges at 2 mV s^–1^ in SIB (inset), and E) normalized contribution ratio of capacitive capacities at different scan rates for the as‐prepared samples.

The discharge/discharge cycling performances of the as‐derived samples are displayed in Figure 6A at 0.1 A g^−1^ between 0.01 and 3.0 V (vs Na/Na^+^). By comparison of 0/1/2/3D electrodes, it is clear that 1D CNF exhibits the best Na‐storage stability in cycling, retaining a reversible capacity of 301.2 mAh g^−1^ after 200 cycles. The discharge capacities were also kept 140 mAh g^−1^ for 0D CQDS (bulk materials), 128.5 mAh g^−1^ for 2D CNS, and 217.4 mAh g^−1^ for 3D CFW. In Figure 8S (Supporting Information), the charge–discharge platforms of different cycles were compared, revealing that the fundamental Na‐storage mechanism has not changed. The capacity fades in the first 20 cycles for all samples, and the following cycles are stable, which is due to the irreversible reaction and the formation of solid electrolyte interphase (SEI) film.^41^ The excellent electrochemical property for 1D CNF was concluded to two reasons: (1) plenty of defects on the surface are beneficial for sodium ions' adsorption/desorption; (2) long‐term disordered graphitize structures with large layer distance provide much insertion entrances. Moreover, the high initial capacity and the low residual capacity of 2D CNS are observed, which is ascribed that large‐scale graphenes are easy to stick together in cycling progress, resulting in the serious capacity fading. It is further demonstrated that 3D CFW with steady framework presents the stable Na‐storage property. In addition, the Coulombic efficiency of 0/1/2/3D electrodes are ≈51%, 53%, 37%, and 21% in Figure 6B and in Figure 10S (Supporting Information), which is attributed to the fact that the irreversible capacity is relative to the variation of special surface area, agreeing well with the previous report.^42^ It is concluded that the special characteristics of 0/1/2/3D samples have significant effects on the cycling stability, which also affect their rate performances. In Figure 6C, the excellent fast Na‐storage properties of 1D CNF is impressive, 309, 272, 221, 183, 145, 117, and 95 mAh g^−1^ at 0.1, 0.2, 0.5, 1.0, 2.5, 5.0, and 10.0 A g^−1^, which is better than the majority of the previous works in **Table** **2**. Meanwhile, 2D CNS and 3D CFW also exhibit the satisfactory transference of sodium ions, delivering large reversible capacities of 118 and 117 mAh g^−1^ at 2.5 A g^−1^. Thus, the platforms at stepwise current densities for 1D CNF are presented in Figure 6D to investigate the surface‐induced capacitive process (SCP) and diffusion‐controlled intercalation process (DIP). The steep platform at 10 A g^−1^ is near to line, while the slopes of small current densities (e.g., 0.1 and 0.2 A g^−1^) are much more mild and arced, further revealing that the total capacity derives from two sources SCP and DIP at low current levels. As well known, the capacity and cycling properties at large current density seriously relied on the SCP behaviors. The rate capacities of 1/2/3D samples at 5.0 A g^−1^ are compared in Figure 6E, displaying stable capacities of 107, 58, and 86 mAh g^−1^ (after 5000 cycles), respectively, which meet the commercial electrode materials. In Figure 11S (Supporting Information), the long‐cycling properties of 1D CNF were displayed, and the capacities can be kept 217.7 mAh g^−1^ at 0.5 A g^−1^ after 500 cycles, 201.2 mAh g^−1^ at 1.0 A g^−1^ after 1000 cycles, and 147.2 mAh g^−1^ at 2.5 A g^−1^ after 2000 cycles, further verifying the excellent electrochemical properties of 1D CNF.

**Table 2:** The comparison of the pure carbon samples

Materials	Raw materials	CE [%]	Cycling/rate [mAh g^−1^]	Year/Ref.
These works	1D carbon fibers	53.36	301.2 at 0.1 A g^−1^ after 200 cycles; 118.5 at 5.0 A g^−1^; 98.2 at 10.0 A g^−1^	
	2D carbon sheets	37.42	128.8 at 0.1 A g^−1^ after 200 cycles; 100.3 at 5.0 A g^−1^; 76.5 at 10.0 A g^−1^	
	3D carbon framework	21.95	217.4 at 0.1 A g^−1^ after 200 cycles; 76.3 at 5.0 A g^−1^; 55.2 at 10.0 A g^−1^	
	Bulk carbon	51.11	140.1 at 0.1 A g^−1^ after 200 cycles	
Commercially hard carbon	Commercially bulk carbon	78		2011/52
Templated carbon	Hierarchical pore system Nanocasting route	20	120 at 0.074 A g^−1^ after 40 cycles; 140 at 0.074 A g^−1^; 100 at 1.85 A g^−1^	2011/53
Hollow carbon nanospheres	Glucose	41.5	160 at 0.1 A g^−1^ after 100 cycles; 100 at 2 A g^−1^; 75 at 5.0 A g^−1^	2012/41
Hollow carbon nanowires	Polyaniline	50.5	≈220 at 0.05 A g^−1^ after 200 cycles; 210 at 0.25 A g^−1^; 149 at 0.5 A g^−1^	2012/22
Carbon nanofibers	Biomass cellulose	58.8	176 at 0.2 A g^−1^ after 600 cycles; 255 at 0.04 A g^−1^; 85 at 2 A g^−1^	2013/54
Macroscopic carbon frameworks	Peat moss	57.5	255 at 0.1 A g^−1^ after 210 cycles 106 at 2.0 A g^−1^; 66 at 5.0 A g^−1^	2013/55
Carbon paper	Commercially carbon paper		137 at 0.1 A g^−1^ after 300 cycles; 100 at 1.0 A g^−1^; 50 at 5.0 A g^−1^	2013/56
Expanded graphite	Graphite	≈49.53	150 at 0.1 A g^−1^ after 2000 cycles 91 at 0.2 A g^−1^	2014/20
Carbon nanofibers	Commercially	36	≈260 at 0.05 A g^−1^ after 280 cycles	2014/57
Banana peel derived pseudographite	Biomass banana peel	67.8	298 at 0.1 A g^−1^ after 300 cycles 100 at 2.0 A g^−1^; 70 at 5.0 A g^−1^	2014/25
Free‐standing porous Carbon nanofibers	Electrospinning process	53.5	266 at 0.05 A g^−1^ after 100 cycles; 164 at 2.0 A g^−1^; 60 at 10.0 A g^−1^	2014/58
Porous carbon/graphene composite	Graphene		250 at 1.0 A g^−1^ after 1000 cycles	2014/59
Carbon microspheres	Sucrose	≈38	183 at 0.03 A g^−1^ after 50 cycles 80 at 1.0 A g^−1^	2014/25
Nanoporous hard carbon	Sugar	≈77	289 at 0.02 A g^−1^ after 100 cycles 95 at 0.5 A g^−1^	2015/60
Natural graphite	Graphite	≈52	127 at 0.1 A g^−1^ after 300 cycles; 103 at 5.0 A g^−1^; 78 at 10.0 A g^−1^	2015/34
Graphite	Graphite	≈93	110 at 0.2 A g^−1^ after 6000 cycles 110 at 10.0 A g^−1^	2015/61
Amorphous carbon/graphene composite	Graphite, glucose		142.0 at 0.5 A g^−1^ after 2500 cycles ≈130 at 5 A g^−1^; 120 at 10 A g^−1^	2015/35
Wood fiber derived carbon	Wood fiber	72	196 at 0.1 A g^−1^ after 200 cycles	2015/62
Honeycomb carbon bubbles	C60 powders	52	209 at 0.1 A g^−1^ after 400 cycles; 112 at 5 A g^−1^	2015/14
3D porous carbon frameworks	CQDs from acetone	34.8	303.2 at 0.1 A g^−1^ after 100 cycles; 104 at 10.0 A g^−1^; 90 at 20.0 A g^−1^	2015/9
Hard carbon from polyvinyl chloride nanofibers	Electrospinning	69.9	215 at 0.012 A g^−1^ after 120 cycles 271 at 0.012 A g^−1^; 147 at 0.24 A g^−1^	2015/44
Hard carbon microtubes	Biomass cotton	83	305 at 0.03 A g^−1^ after 100 cycles; 180 at 0.3 A g^−1^	2015/63
Hard carbon nanoparticles	Polyaniline	51.6	207 at 0.05 A g^−1^ after 500 cycles	2015/37
Few‐layered graphene (solvent‐assisted intercalation)	Chemical vapor deposition processes	≈58	≈115 at 12 A g^−1^ after 8000 cycles; 125 at 10 A g^−1^; 100 at 30.0 A g^−1^	2016/64
Microporous spherical carbon	Furfuryl alcohol	67.3	232 at 0.02 A g^−1^ after 40 cycles; 67 at 1.0 A g^−1^	2016/65
Corn cobs derived carbon	Biomass corn cobs	86	275 at 0.06 A g^−1^ after 100 cycles; 211 at 0.6 A g^−1^	2016/36
Apple biowaste derived hard carbon	Biomass apple biowaste	61	230 at 0.02 A g^−1^ after 80 cycles; 112 at 2.0 A g^−1^	2016/66
Carbonized‐leaf membrane	Biomass leaf	74.8	270 at 0.04 A g^−1^	2016/67
Holly leaf derived lamellar carbon	Betula platyphylla and Sophora japonica	60	253 at 0.02 A g^−1^ after 1000 cycles; 103 at 0.2 A g^−1^	2016/45
Disordered 3D multilayer graphene	Graphene	58.6	100 at 0.75 A g^−1^ after 500 cycles; 118 at 0.75 A g^−1^	2016/68
Mesoporous soft carbon	Mesophase pitch	45	≈200 at 0.05 A g^−1^ after 200 cycles; 62 at 5.0 A g^−1^	2016/69
Pitch and lignin derived carbon	Pitch and lignin	82	226 at 0.03 A g^−1^ after 150 cycles; 162 at 0.3 A g^−1^	2016/26
Rod‐like ordered mesoporous carbons	Triblock copolymer/silica/glycerol	71.26	159.3 at 0.1 A g^−1^ after 100 cycles; 120 at 1.0 A g^−1^	2016/51
Polydopamine derived carbon	Polydopamine	53.7	508 at 0.05 A g^−1^ after 1000 cycles; 122 at 3.2 A g^−1^	2016/70
Rape seed shuck derived hard carbon	Biomass rape seed shuck	≈80	143 at 0.1 A g^−1^ after 200 cycles; 32 at 5.0 A g^−1^	2017/71
3D hollow reticulate hard carbon	Rape pollen grains		145 at 0.1 A g^−1^ after 400 cycles; 50 at 2.0 A g^−1^	2017^72^
Quinone molecules encapsulated in single‐walled carbon nanotubes (SWCNTs)	Commercially SWCNTs		≈200 at 0.1 A g^−1^; ≈180 at 0.8 A g^−1^	2017^73^
3D neat porous carbon aerogels	Polymerization of ligin, resorcinol and formaldehyde	41	297 at 0.05 A g^−1^ after 100 cycles; 139 at 1.0 A g^−1^	201751
Carbon network	Biomass kiwifruit	55.8	426 at 0.1 A g^−1^ after 100 cycles; 182.6 at 2 A g^−1^	2017^74^
Bulk carbon	Biomass dandelion	59.4	372 at 0.05 A g^−1^ after 300 cycles	2017^75^
Graphdiyne	Hexaethynylbenzene	52.8	380 at 2.5 A g^−1^	2017^76^
Hard carbon	Sucrose		287 at 0.1 A g^−1^ after 150 cycles; 85 at 0.6 A g^−1^	2017^77^
Hierarchically nanoporous pyropolymer nanofibers	Electrospinning		245 at 0.1 A g^−1^; 143 at 5 A g^−1^	2017^78^
Nanosheets	White sugar	55.77	423 at 0.25 A g^−1^ after 150 ≈160 at 1.0 A g^−1^	2017^79^
Graphene/carbon nanotube hybrid	Urea, GO		269 at 0.3 A g^−1^ after 100; 177 at 5.0 A g^−1^	2017^80^
Hard carbon	Biomass argan shells	79.0	295 at 0.025 A g^−1^ after 60 cycles	2017^81^
Mesoporous carbon	Coconut shells		≈160 at 0.1 A g^−1^ after 70 cycles	2017^82^
3D carbon	polyaniline (PANI), GO	49	323 at 1.0 A g^−1^ after 1000 cycles; 218 at 5.0 A g^−1^	2017^83^
Carbon sheets	Wheat straws	50.53	221 at 0.05 A g^−1^ after 200 cycles; 109 at 2.0 A g^−1^	2017^18^
Hard carbon	Orange peel		156 at 0.5 A g^−1^ after 100 cycles; 117 at 1 A g^−1^	2017^84^
Hard carbon	Mangosteen shell		303.6 at 0.02 A g^−1^ after 100 cycles; 85 at 0.2 A g^−1^	2017^85^
3D hierarchical mesocarbon microbead	Graphite		177.5 at 0.1 A g^−1^ after 100 cycles; 121 at 0.8 A g^−1^	2017^86^
Carbon fibers	Electrospinning	67	249.6 at 0.1 A g^−1^ after 100 cycles; 40 at 1.0 A g^−1^	2017^87^
Porous carbon spheres	d‐Glucose	50	172 at 0.5 A g^−1^ after 1000 cycles; 122.2 at 2.5 A g^−1^	2017^88^
Porous carbon	Glucose		300 at 0.1 A g^−1^ after 400 cycles; 220 at 0.2 A g^−1^	2017^89^

In view of this intriguing structure evolution for 1D CNF, the variations of electrochemical properties were carried out as presented in **Figure** **7**. The cycling stability of CNF‐400/500/600/700 at 0.2 A g^−1^ was displayed in Figure 7A. Due to the excellent structural features (e.g., high degree of crystalline lattice and long‐term disordered lattice), CNF‐700 shows a reversible capacity of 240.9 mAh g^−1^ after 200 cycles, larger than those of CNF‐400/500/600 (128.3, 211.7, and 228.7 mAh g^−1^). Figure 7B displays the initial discharge–charge platform curves of CNF‐700, and it possesses the discharge–charge capacities of 535 and 274.9 mAh g^−1^ with a coulombic efficiency (CE) of 51.4%. Although the first sodiation–desodiation capacities were found for other electrodes, the lower CE values were obtained as 30.3%, 42.1%, and 40.9%, mainly derived from different special surface areas in Figure 12S (Supporting Information). Moreover, with the improved annealing temperatures, the voltage platforms were lowered in the order of CNF‐400 (1.24 V) > CNF‐500 (1.01 V) > CNF‐600 (0.94 V) > CNF‐700 (0.75 V), which are beneficial for the enlarged energy density. Note that the largest CE and lowest voltage platforms of CNF‐700 are crucial for the practical applications. Clearly, the outstanding rate performance (111.7 mAh g^−1^ at 5.0 A g^−1^) can be noticed for CNF‐700, which is ascribed to the introduction of effective defect, the expanded lattice space, and the increased lattice domain, facilitating the ion/electrons diffusion.^43^ In addition, it is noteworthy that their following cycling is still stable at 1.0 A g^−1^, demonstrating the best reversible properties. Even at a large current density of 2.0 A g^−1^, the satisfactory cyclic stability of CNF‐700 (158.9 mAh g^−1^) is exhibited after 2000 cycles, obviously higher than those of other electrodes.

According to the aforementioned discussions, numerous differences on the characteristics were found for 1/2/3D samples from the evolution of 0D CQDs, further giving rise to the distinctions of the electrochemical kinetics. In Figure 13S (Supporting Information), the disappeared reduction peaks at first curves are due to the formation of SEI film with the decomposition of the electrolyte.^44^ After the first charge/discharge process, it is clear that the second, third, and fourth curves are overlapped, indicating that the stable SEI film is accomplished. Through the assistances of the cyclic voltammetry (CV) curves at different scan rates, two types of energy storage modes (SCP and DIP) were analyzed and the corresponding ratios were quantified. In **Figure** **8**A–C, three couples of peaks for the as‐derived samples were clearly observed between 0.01 and 0.5 V (vs Na/Na^+^), and they are sharper in the order of CNF‐400 < CNF‐500 < CNF‐600 < CNF‐700, and 1D‐CNF‐700 > 2D > 3D, indicating the reduction of sodium storage in the graphite layers, which is ascribed to the variation of lattice interlayer in Table 1.^45^ These further demonstrate that the sodium ions insert/extract into graphite layers or microspores at low potential, which is consistence with the previous work.[[qv: 25b,46]] At low potential, the oxidation peak of 1D CNF‐700 is clearly sharper than that of 2/3D samples and that is gradually enhanced from CNF‐400 to CNF‐700 in Figure 8A–C. It is reasonable that the ordered graphitization lattice with the largest interlayer would provide numerous sodium‐insertion channels, favoring the electrochemical performances.^35^ It is interesting to note that all the peak shapes of the samples are nearly identical even through the voltages rising from 0.1 to 9.0 mV s^−1^, verifying small polarization at high rate current densities in Figure 8 and Figure 14S (Supporting Information).^47^ Based on Dunn and co‐workers' work,^48^ the charge/discharge mechanism and the content of capacitive contribution can be quantified analyzed by Equations (1) and (2)

(1) $$i\;=\;av^{b}$$

(2) $$i\left(V\right)\;=\;k_{1}v\;+\;k_{2}v^{1/2}$$

Considering Equation (1), the *b*‐value close to 1 implies SCP, while that approaching 0.5 indicates DIP.^35^ The fitting lines and *b*‐values are shown in Figure 8D, revealing the stable electrochemical behaviors and enhanced capacitance‐controlled ratios in sequence: CNF‐400 < CNF‐500 < CNF‐600 < CNF‐700. Obviously, the largest *b*‐value (0.84) is found for 1D CNF‐700, illustrating that 1D CNF with most effective defects and largest lattice space was favored by SCP.^49^ The effective defects could render more sodium‐ion storage, and the large lattice distance could reduce the resistance of sodium ions' insertion/extraction, which is important for SCP behaviors. To further investigate the ratio of SCP and DIP for the total capacity at different scan rates, Equation (2) is utilized, and *k*_1_ *v* and *k*_2_ *v* ^1/2^ are associated with SCP and DIP, respectively. In the inset of Figure 8D and in Figure 15S (Supporting Information), the largest ratio of SCP for 1D CNF‐700 samples is 87% at 0.9 mV s^−1^, which matches well with the results of the slope. In addition, the ratios of SCP increase at mounting scan rates, further conforming that the electrochemical properties are determined by SCP.^50^ Thus, the strongest SCP was found for 1D CNF, which is corresponding to the best rate performances. Two reasons are deduced to explain the high SCP for 1D CNF: (1) much sodium ions' insertion entrances are provided by partial‐ordered graphitize structures with large layer distance; (2) the existence of effective defects and micropores facilitates the reversible Na‐adsorption/desorption on the surface.^35^

**Figure 9:**
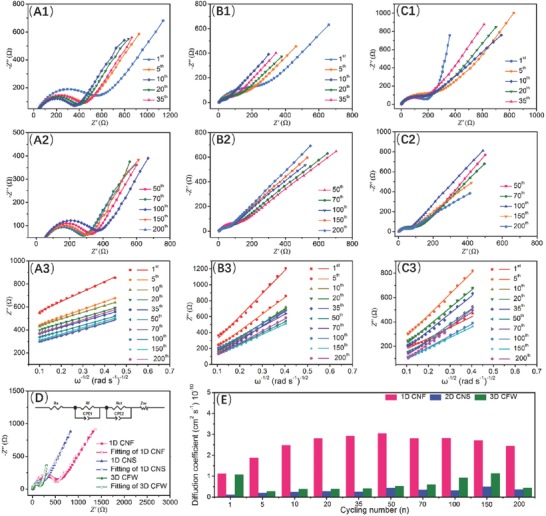
Nyquist plots at discharge stage (0.8 V) after different cycling, the fitting lines in the low frequency for A) 1D CNF, B) 2D CNS, C) 3D CFW. D) Impedance spectra and fitting curve using the equivalent circuit (inset). E) Sodium diffusion coefficients after various cycling for 1/2/3D samples.

To better understand the Na‐storage behaviors at different cycles for the as‐prepared samples, electrochemical impedance measurements (EIS) was conducted to confirm the corresponding sodium‐ion coefficients (*D*_Na+_). The resistances at undischarged condition are exhibited in Figure 16S (Supporting Information), where it is found that of 3D CFW is much larger than that of 1/2D samples and the resistance is decreasing for CNF‐400/500/600/700. As shown in **Figure** **9**, two depressed semicircles appeared in the middle–high frequency regions, while a linear slope located at the low‐frequency region in all impedance spectra, revealing the charge transfer resistance and Warburg diffusion of sodium ions in the internal of samples, respectively. Based on these commons features, the equivalent circuit (the inset of Figure 9D) was established to explore various components of impedance. This can be divided into four parts: (1) *R*_e_ is related to the resistance of the materials in cell; (2) *R*_f_ and *CPE1* are associated with the SEI films; (3) *R*_ct_ and *CPE2* are equivalent to the charge transfer resistance; and (4) *Z*_w_ as the important criterion for evaluating the speed of Na‐transfer is presented by the linear slope. It is found that the total of *R*_ct_ and *R*_f_ for 1D CNF is slight larger than that of 2D CNS, 3D CFW, which is ascribed that the high degree of graphitization would improve the electronic conductivity, giving rise to the small resistance. Moreover, the impedances of all the samples were decreased gradually with the increasing charge/discharge cycles, suggesting the decreasing resistances of electrode, which are beneficial for electron transferring. According to the Warburg factor related to *Z*_w_ (Equations (3) and (4)), the *D*_Na_ *_+_* of all the curves was quantified and analyzed

(3) $$Z_{w}\;=\;R\;+\;\sigma \omega^{-1/2}$$

(4) $$D\;=\;R^{2}T^{2}/2S^{2}n^{4}F^{4}C^{2}\sigma^{2}$$

The fitting lines about *Z′* versus ω^−1/2^ (*ω = 2πf*) were displayed in Figure 10S in the Supporting Information (A3, B3, C3), to further obtain σ. Obviously, the slopes of 1D CNF are smaller than those of other samples, and the larger the σ, the smaller the *D*_Na+_. In addition, the *R* in Equation (4) is the gas constant (8.314 J mol^−1^ K^−1^); *T*, *S*, *F*, *C* are the absolute temperature (297.15 K), the electrode surface area (1.15 cm^2^), the Faraday constant (96 485 A mol^−1^), and the concentration of electrolyte (1 mol L^−1^) respectively; and *n* is obtained from the equation (*Q* = *nF*/3.6*Mr*). Note that *D*_Na+_ of 1D CNF is much higher than that of 2/3D samples at various cycles in Figure 9E. For examples, from Table 2S (Supporting Information), the *D*_Na+_ of 1D CNF at 100 cycles is 2.81 × 10^−10^ cm^2^ s^−1^, about nine times than that of 2D CNS (0.32 × 10^−10^ cm^2^ s^−1^) and three times than that of 3D CFW (0.91 × 10^−10^ cm^2^ s^−1^), which facilitates the Na transfer in the internal of carbon samples.^37^, ^51^

## 3 Conclusion

In summary, controlling 1D CNF, 2D CNS, and 3D CFW is effectively addressed via the aid of temperature‐induced intermediates of salts and the self‐assembly of 0D CQDS. The catalytic characteristics of the reactant production (ZnO, Ni, and Cu) are vital for the in situ construction of multidimensional carbon samples through the “orient induction” of ZnO, the “dissolution–precipitation” of Ni, as well as the “surface adsorption self‐limited” of Cu, further revealing that lattice spaces, the degree of graphitization, effective defects, and special surface area can be effectively manipulated. The formation mechanism of 1D CNF was further explored and deemed as the “vines' style” growth mechanism triggered by the high energy from the improved temperature. As expected, thanks to theses structure advantages, when 1/2/3D samples are used as anodes for SIBs, the electrochemical properties were greatly enhanced. 1D CNF shows the high reversible capacity of 324.9 mAh g^−1^ at 0.1 A g^−1^, and it retains 301.2 mAh g^−1^ after 200 cycles, as well as the excellent capacity (107 mAh g^−1^ at 5.0 A g^−1^ after 5000 cycles). Moreover, 2D CNS and 3D CFW display the good rate performance, delivering Na‐storage capacities of 92.2 and 90.9 mAh g^−1^ at 5.0 A g^−1^ after 3000 cycles. The pseudocapacitive contributions are quantificationally analyzed to confirm the fundamental point of the fast Na^+^ storage for 1D CNF. The construction of multidimensional carbon samples from the catalytic features shed light on the preparation of carbon structure and wide application.

## 4 Experimental Section

All the chemicals in this work were obtained from the Aladdin and used without any purification.

*Preparation of 0D CQDs*: In this typical process, some modifications were carried out. With the magnetic stirring (400 rad min^−1^), 12 g of NaOH (Alpha) was reacted with 40 mL of acetone (Alpha) for 1 h. Then, the mixture was kept for 200 h in dry environment under ambient temperature and pressure. Through the acid leach with 1 m HCl solution, the neutral product was obtained, further centrifuged, and washed with deionized water for five times to get the final product. After drying for 24 h to remove the water, the CQDs' powder was achieved.

*Preparation of 1D CNF, 2D CNS, and 3D CFW*: The salts (ZnCl_2_, NiCl_2_, and CuCl) (Alpha) and CQDs were mixed thoroughly via grinding in an agate mortar with the ratio of 10:1 in weight. The as‐prepared mixtures were calcined in a one‐tube furnace under Ar atmosphere at 700 °C (400/500/600 °C) for 5 h, with a heating rate of 10 °C min^−1^. The mixture accompanied with ZnCl_2_ was washed by magnetic stirring and ultrasonication with 1 m HCl solution for 12 h, further conducting suction filtration with water and ethanol washing to obtain 1D structures. The mixture with NiCl_2_ was placed in the concentrated hydrochloric acid and stirred at 60 °C for 12 h, then washed with water and ethanol to neutralize and to get 2D sheets. The mixture containing CuCl was washed with FeCl_3_ and 1 m HCl, further achieving the final product with water and ethanol. All the products were dried in a vacuum at 100 °C for over 12 h to prepare a 3D framework.

*Materials' Preparation*: A power X‐ray diffractometer (Rigaku D/max 2550 VB+18 kW, Japan (Cu Kα radiation, λ = 0.1546 nm, *V* = 40 kV, *I* = 30 mA)), a Laser Micro‐Raman spectrometer (Renishaw InVia, UK), an X‐ray photoelectron spectrometer (K‐Alpha 1063, UK) were used to characterize the crystalline structure of the as‐obtained samples. X‐ray photoelectron spectroscopy (XPS) (K‐Alpha 1063) and FTIR (AVTA‐TAR, 370) were used to analyze these functional groups. The surface morphologies and internal structure were investigated through SEM (a JEM‐2100F instrument at 200 kV) and TEM.

*Electrochemical Tests*: In a MBraun glove box, the CR‐2016 cells accompanied with Na metal (purity, 99.5%) as counter electrode were assembled to test the electrochemical properties of the as‐obtained samples with argon concentrations of moisture and oxygen below 5 ppm. The active mass of every electrode was about 0.7–1.0 mg. The electrolytes were 1 m NaClO_4_ in fluoroethylene carbonate: propylene carbonate) with the ratio of 95:5. Polypropylene (Celgard 2400) was used as a separator. Land CT 2001 battery and Arbin battery cycler (BT2000) were used to investigate the galvanostatic charge/discharged and rate performances at a series of current densities in the voltage window of 0.01–3.0 versus Na/Na^+^. EIS and CV) were carried out using the MULTI AUTOLAB M204 instruments under various conditions.

## Conflict of Interest

The authors declare no conflict of interest.

## Supporting information

Supplementary
