# Supplementary material for: Multidimensional Evolution of Carbon Structures Underpinned by Temperature‐Induced Intermediate of Chloride for Sodium‐Ion Batteries
Source: Adv Sci (Weinh). 2018 Mar 25;5(6):1800080. doi: 10.1002/advs.201800080 (PMC6010011; doi:10.1002/advs.201800080)
Supplement: Supplementary file 1 — Supplementary [file ADVS-5-1800080-s001.pdf]

Copyright WILEY-VCH Verlag GmbH & Co. KGaA, 69469 Weinheim, Germany, 2018.

ADVANCED SCIENCE
Open Access

Supporting Information

for *Adv. Sci.*, DOI: 10.1002/advs.201800080

Multidimensional Evolution of Carbon Structures Underpinned by Temperature-Induced Intermediate of Chloride for Sodium-Ion Batteries

*Peng Ge, Hongshuai Hou, Xiaoyu Cao, Sijie Li, Ganggang Zhao, Tianxiao Guo, Chao Wang, and Xiaobo Ji**

Copyright WILEY-VCH Verlag GmbH & Co. KGaA, 69469 Weinheim, Germany, 2016.

Supporting Information for

Multi-Dimension Evolution of Carbon Structures Underpinned by Temperature Induced Intermediate of Chloride for Sodium-Ion Batteries

Peng Ge, Hongshuai Hou, Xiaoyu Cao, Sijie Li, Ganggang Zhao, Tianxiao Guo, Chao Wang and Xiaobo Ji *

Dr. P. Ge, Prof. Dr. H. Hou, S. Li, G. Zhao, T. Guo and Prof. Dr. X. Ji*
State Key Laboratory of Powder Metallurgy, College of Chemistry and Chemical Engineering, Central South University, Changsha, 410083, China
E-mail: xji@csu.edu.cn

Prof. Dr. X. Cao.
College of Chemistry, Chemical and Environmental Engineering, Henan University of Technology, Zhengzhou, 450000, China

Prof. Dr. C. Wang
School of Energy Science and Engineering, University of Electronic Science and Technology of China, Chengdu, 611731, China

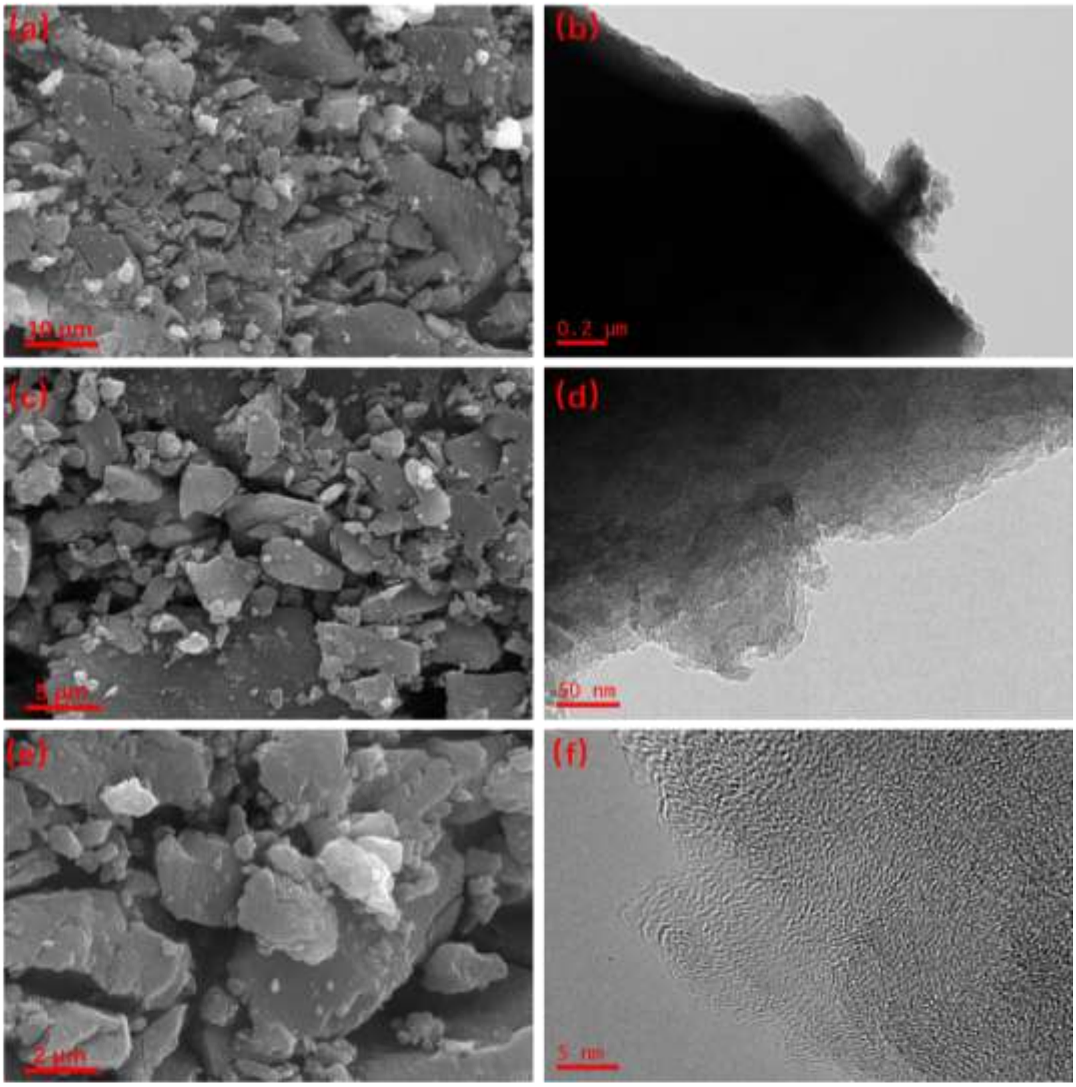

Figure.1S SEM and TEM images of carbon particles (the carbonization of CQDs without any salts).

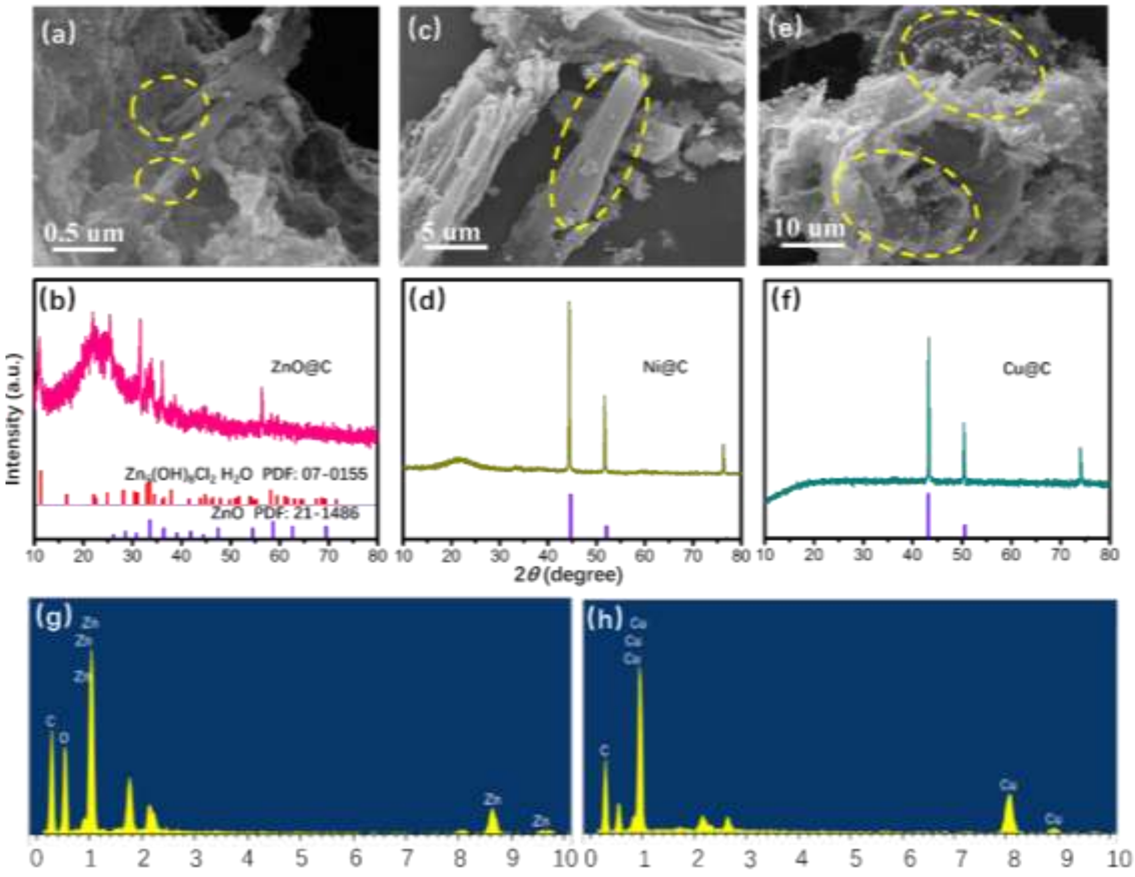

Figure. 2S SEM images of the intermediate mixture and the corresponding XRD of 1D CNF (a, b), 2D CNS (c, d), 3D CFW (e, f), the mapping of the intermediate mixture 1D CNF (g), 3D CFW (h).

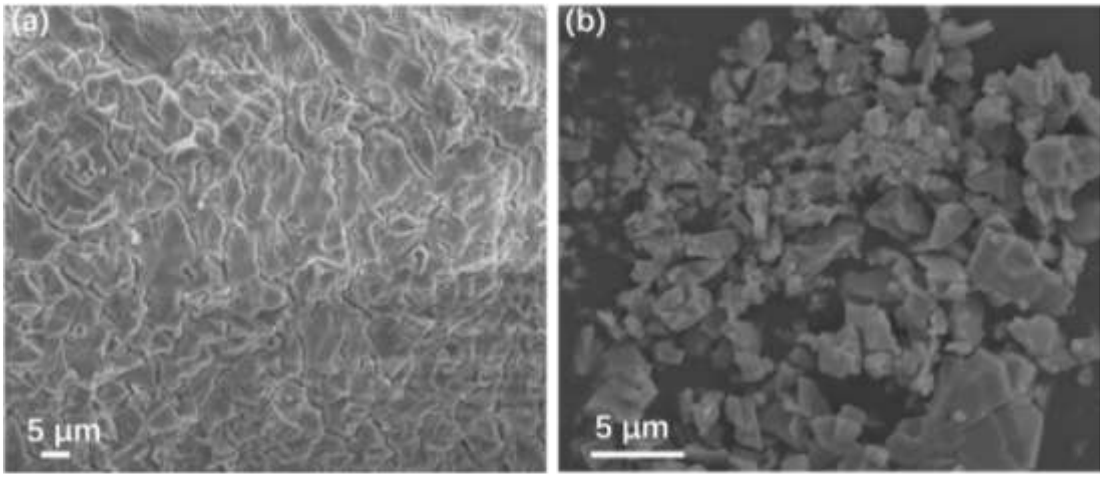

Figure. 3S SEM images of $NiCl_2$ (a), CuCl(b).

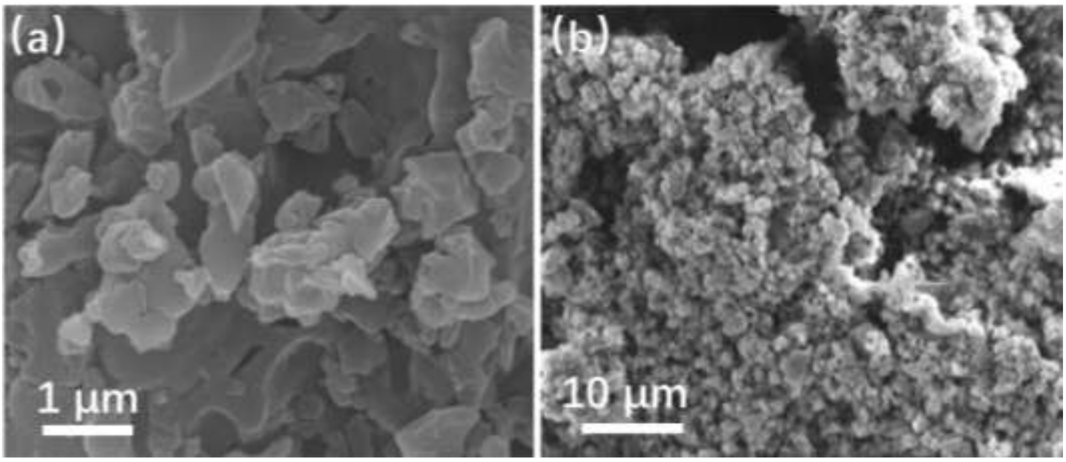

Figure. 4S SEM images of 0D CQDs (a) and 0D CQDs with $ZnCl_2$ at 300 ^oC (b).

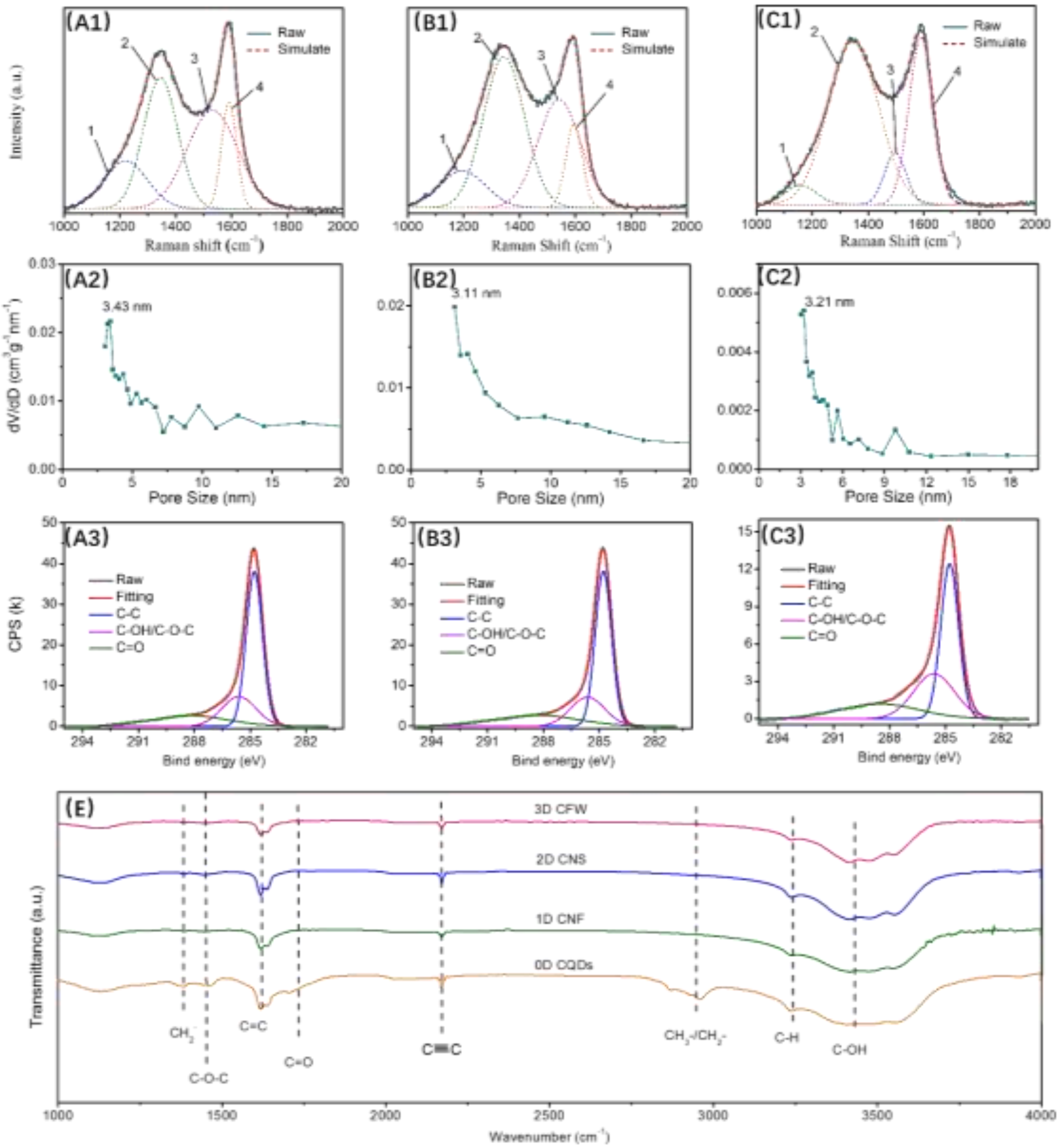

Figure. 5S Raman spectroscopy, the pore size distribution and X-ray photoelectron spectroscopy (XPS) spectra of C 1s spectra of 1D CNF (A), 2D CNS (B) and 3D CFW (C), FTIR (E) of the as-derived samples and CQDs, 1D CNF, 2D CNS, 3D CFW.

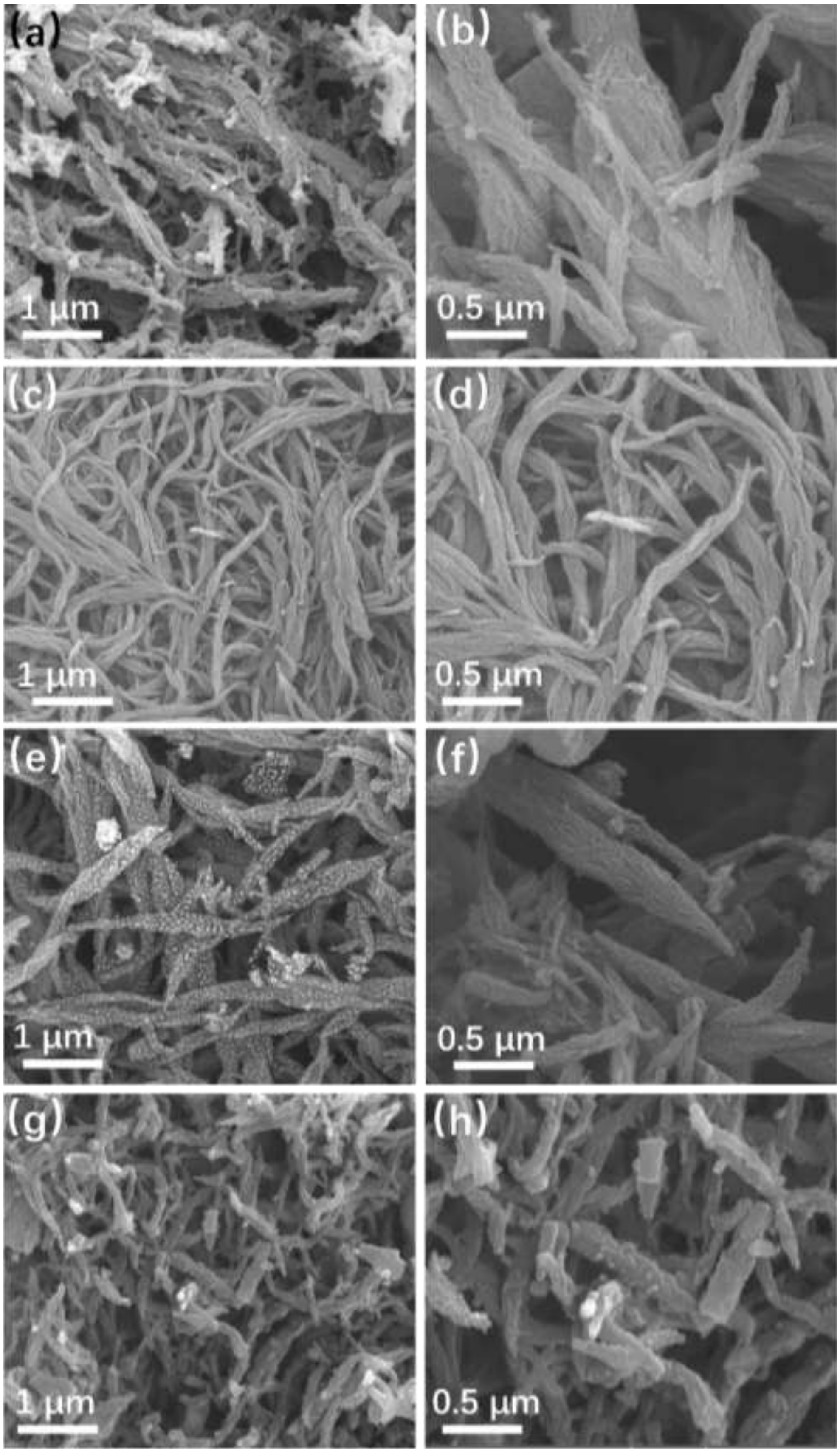

Figure. 6S SEM images of CNF-400 (a, b), CNF-500 (c, d), CNF-600 (e, f), CNF-700 (g, h).

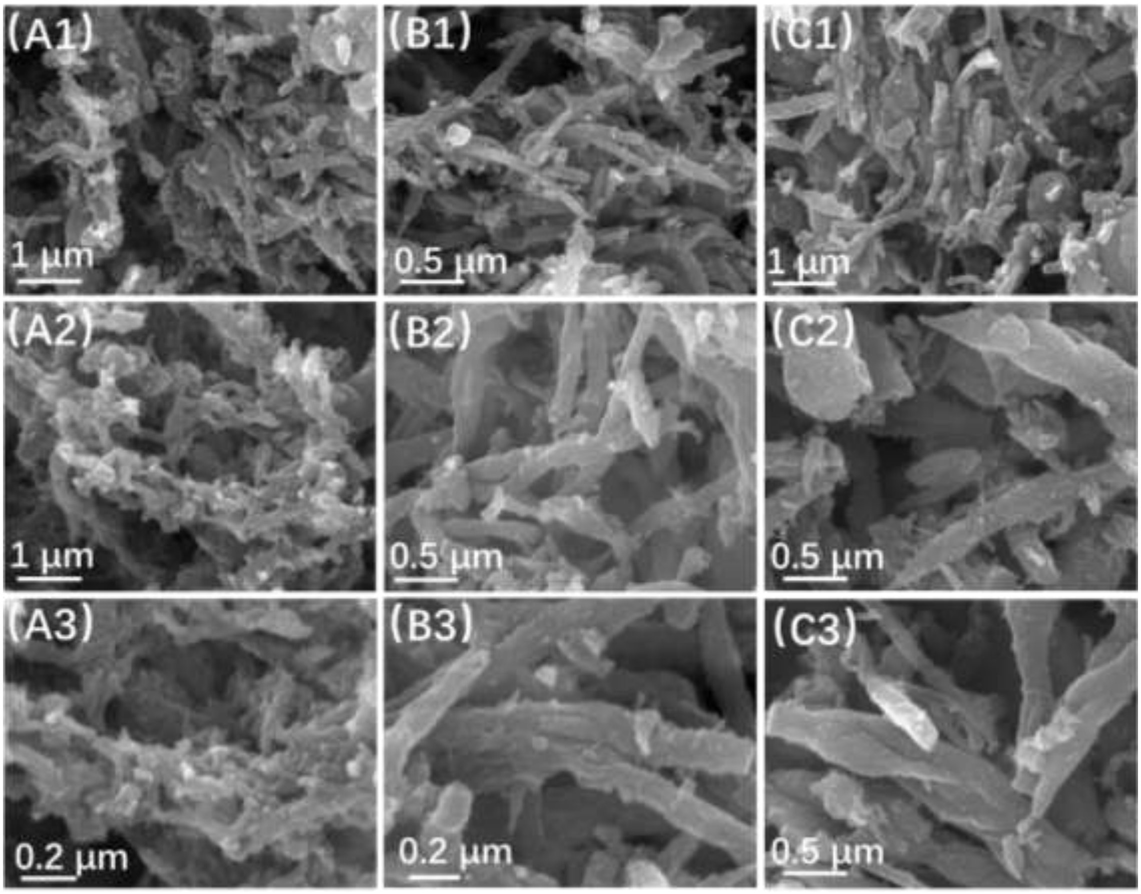

Figure. 7S The ratio of $ZnCl_2$: 0D CQDs, 5 : 1 at 700 ^oC for 5 h (A), 10 : 1 at 700 ^oC for 5 h (B), 10 : 1 at 700 ^oC for 10 h (C).

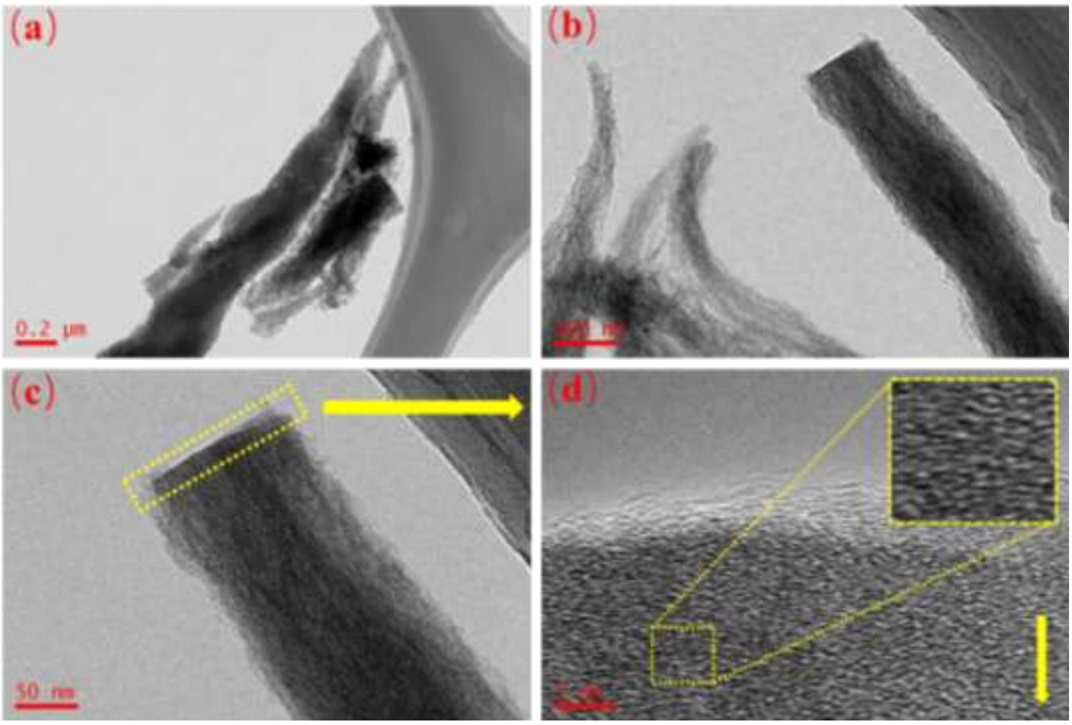

Figure. 8S The TEM and HRTEM images of the root for CNF-700.

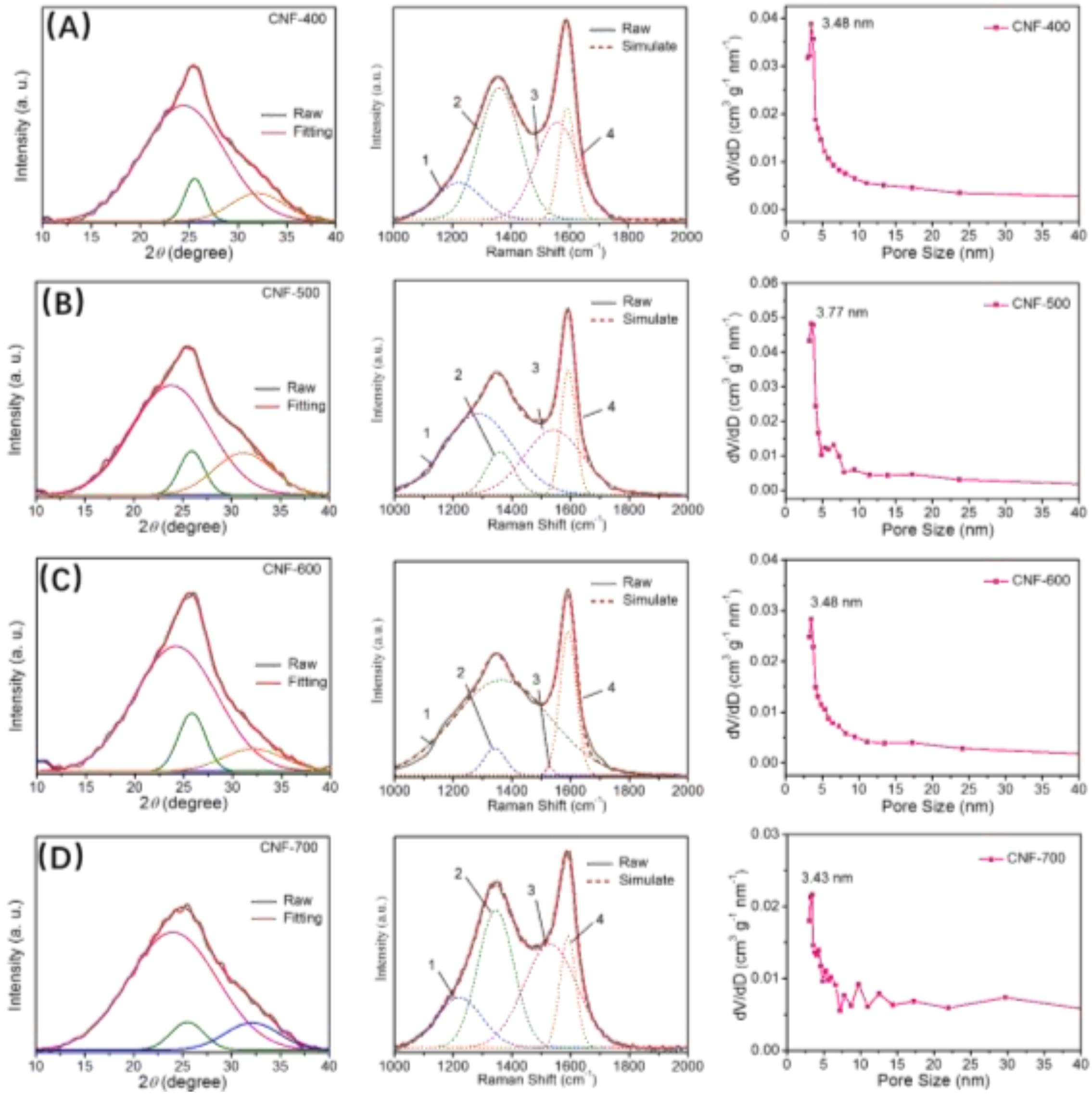

Figure. 9S XRD, Raman and pore distribution of CNF-400 (A), CNF-500 (B), CNF-500 (C) and CNF-700 (D).

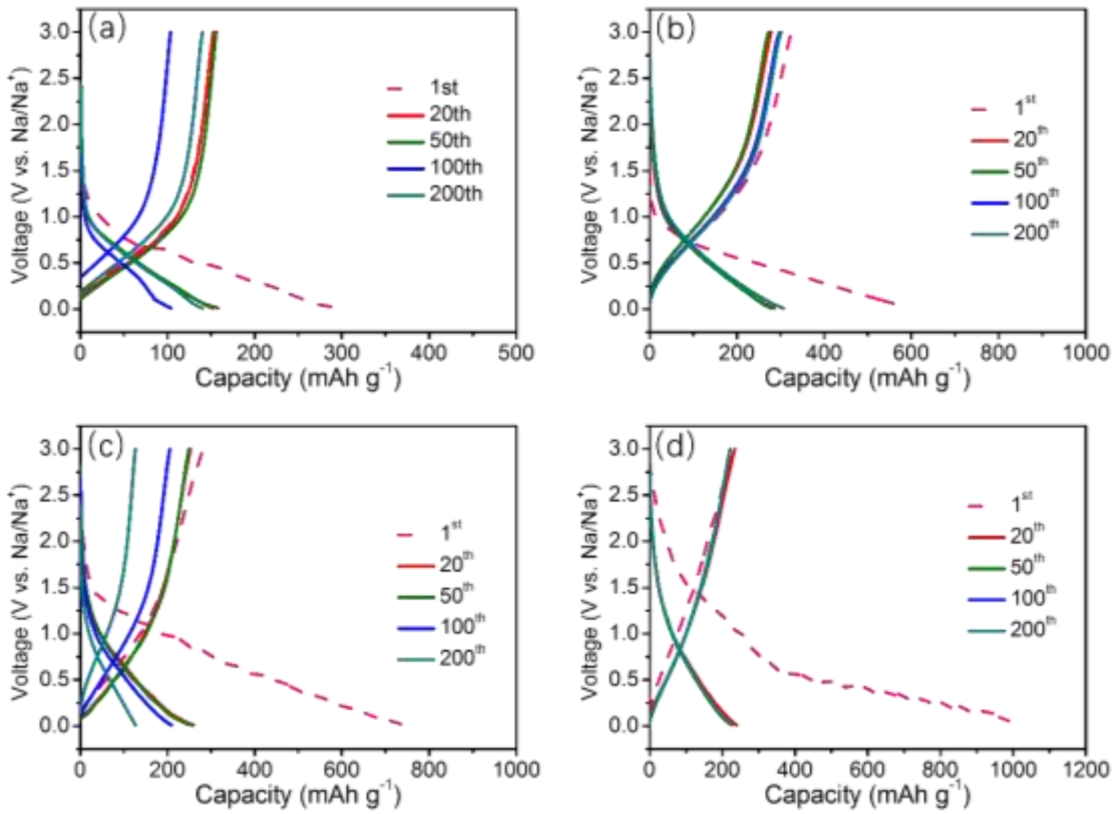

Figure.10S The galvanostatic charge–discharge profiles of 0D CQDs, 1D CNF, 2D CNS, 3D CFW.

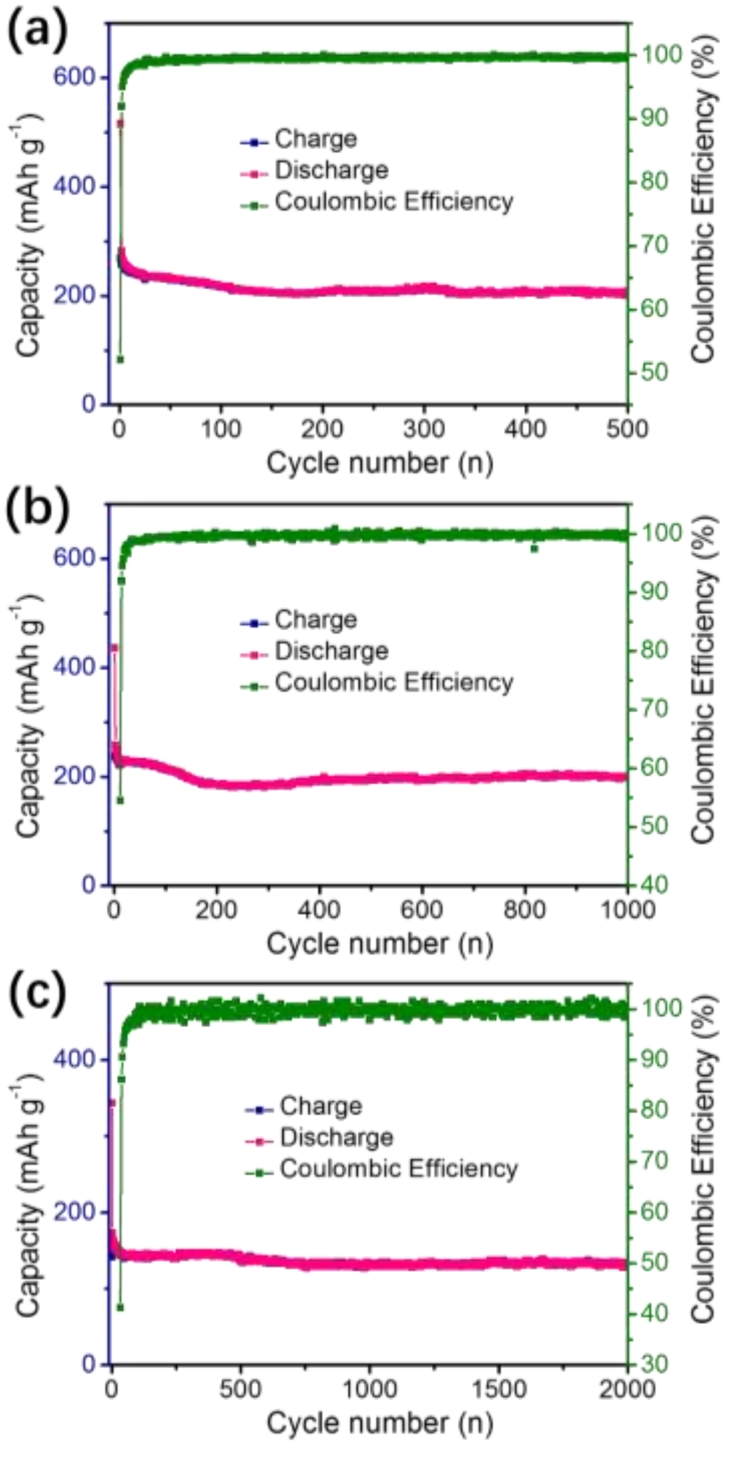

Figure. 11S The galvanostatic charge–discharge cycling of1D CNF at 0.5 A g^{-1}, 1.0 A g^{-1}, 2.5 A g^{-1}.

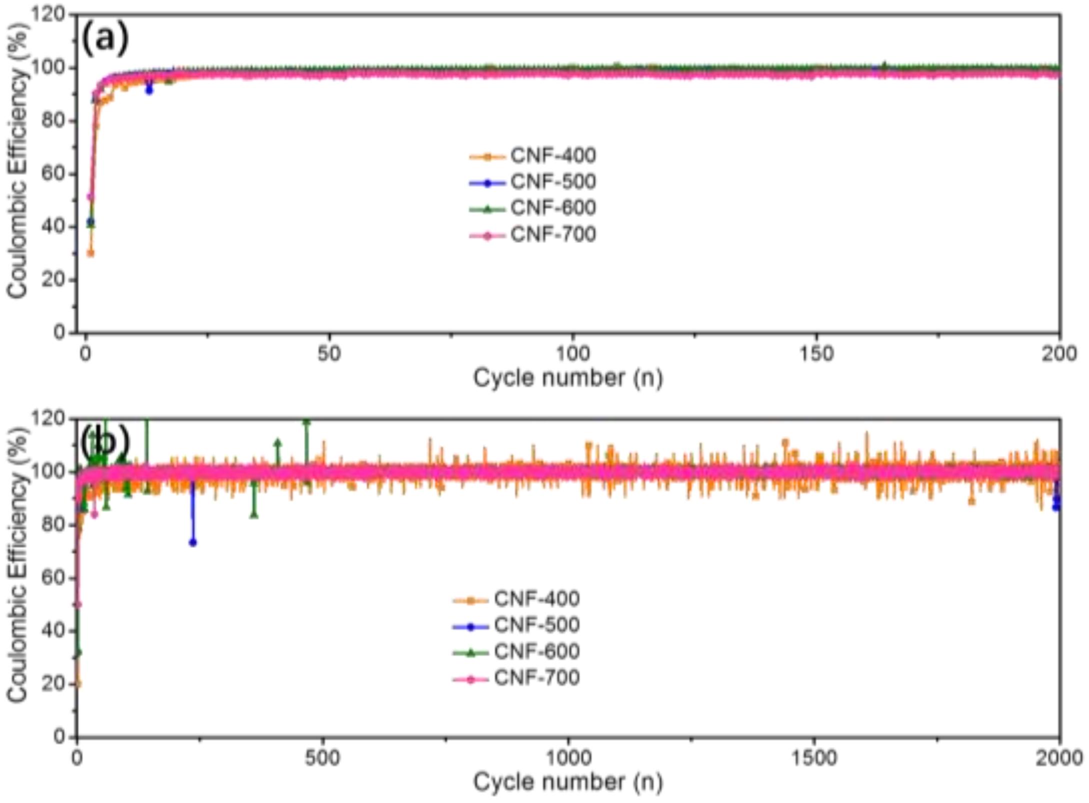

Figure. 12S Coulombic Efficiency of CNF-400/500/600/700, at 0.2 A g^{-1} (a), 2.0 A g^{-1} (b).

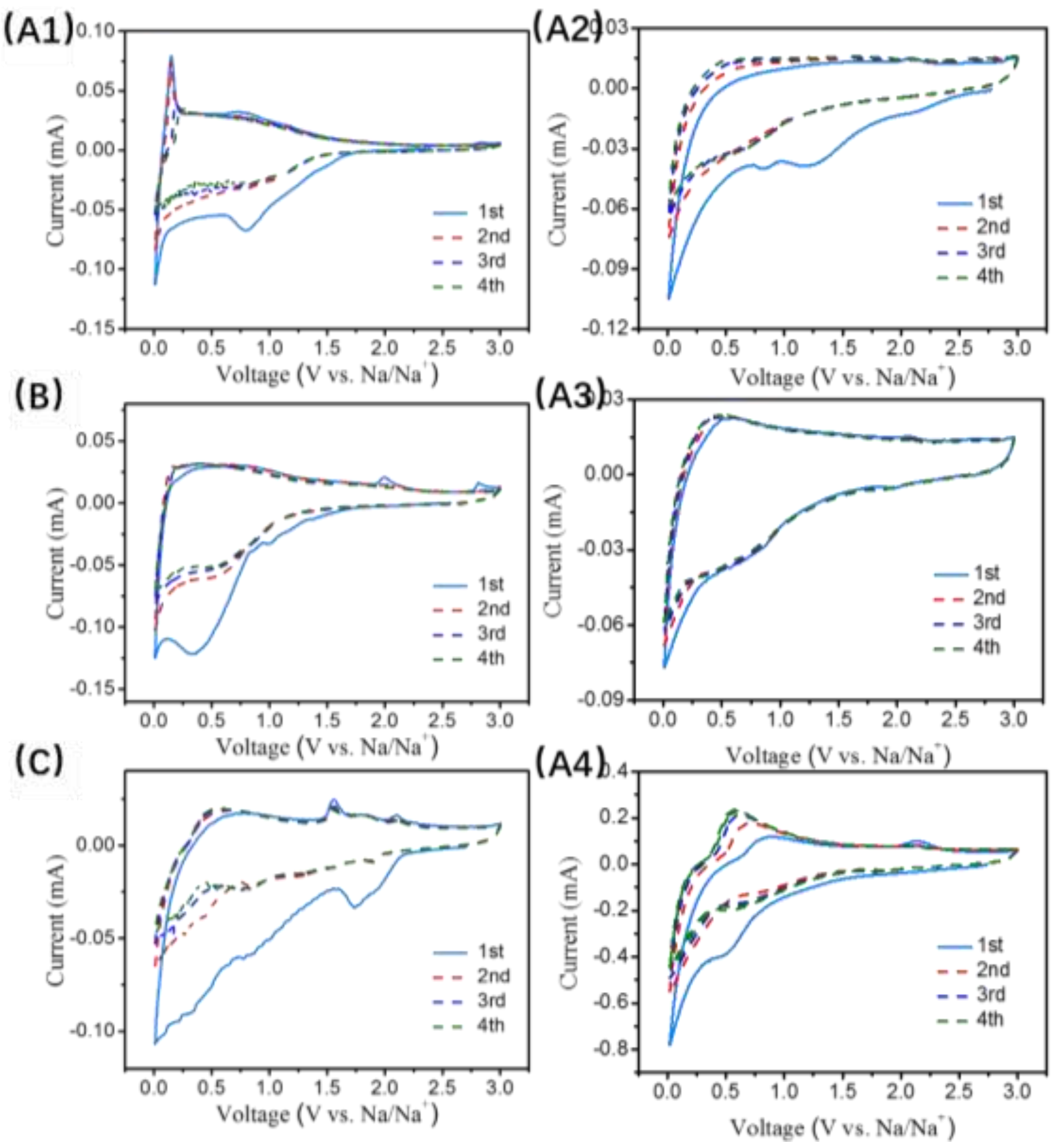

Figure. 13S The first four CV curves at 0.1 mV s^{-1} for 1D CNF (A), 2D CNS (B), 3D CFW (C), CNF400/500/600 (A2, A3, A4).

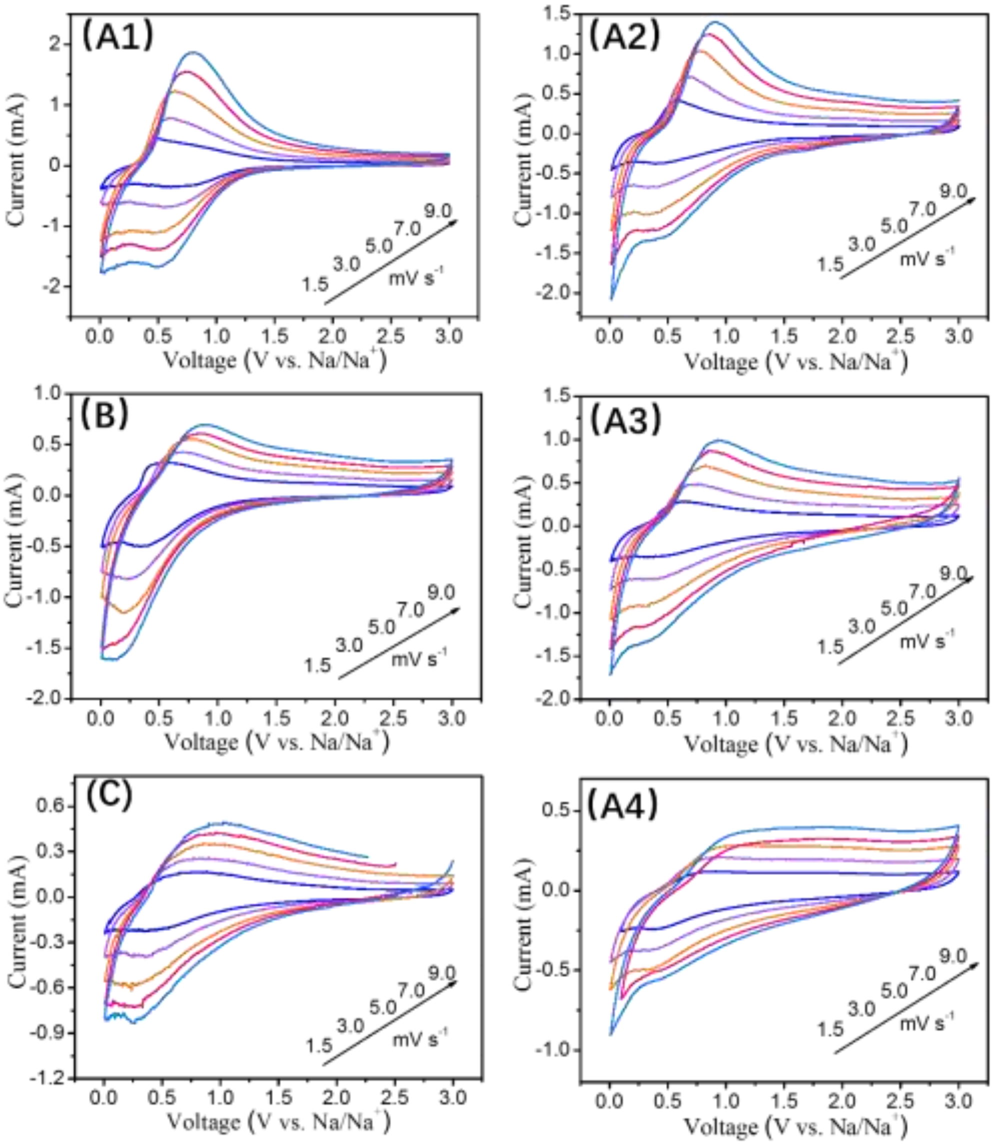

Figure. 14S The first four CV curves at large scan rates for 1D CNF (A), 2D CNS (B), 3D CFW (C), CNF400/500/600 (A2, A3, A4).

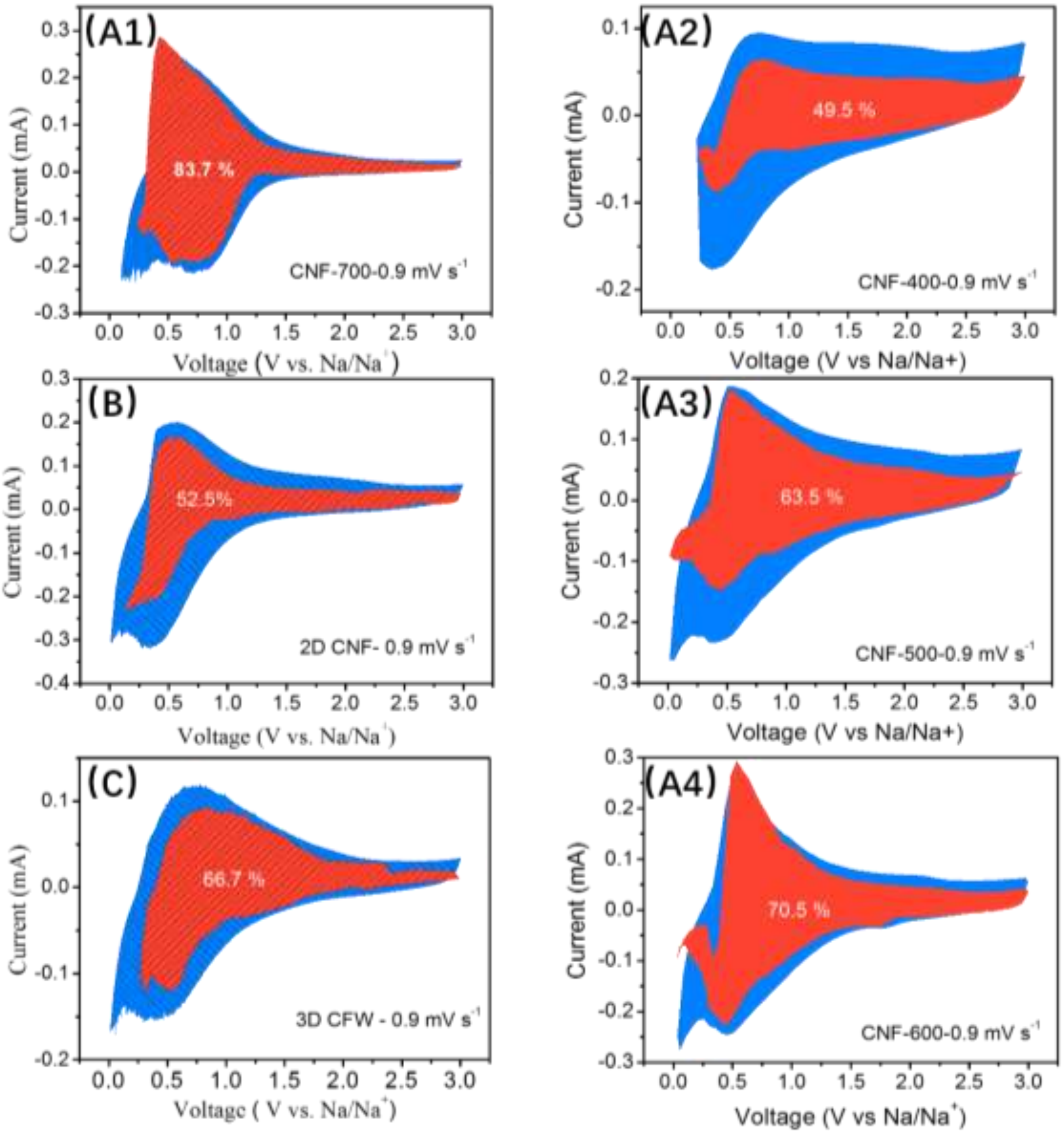

Figure. 15S Separation of the capacitive and diffusion-controlled charges at 2 mV s^{-1} in SIB for 1D CNF (A), 2D CNS (B), 3D CFW (C), CNF400/500/600 (A2, A3, A4).

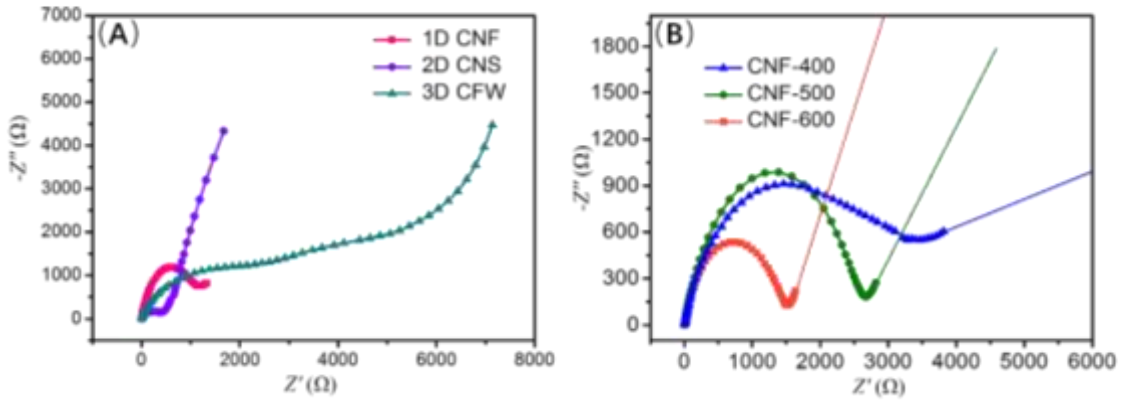

Figure. 16S The Nyquist plots at undischarged condition for multi-dimension samples (A) and CNF-400/500/600 (B).

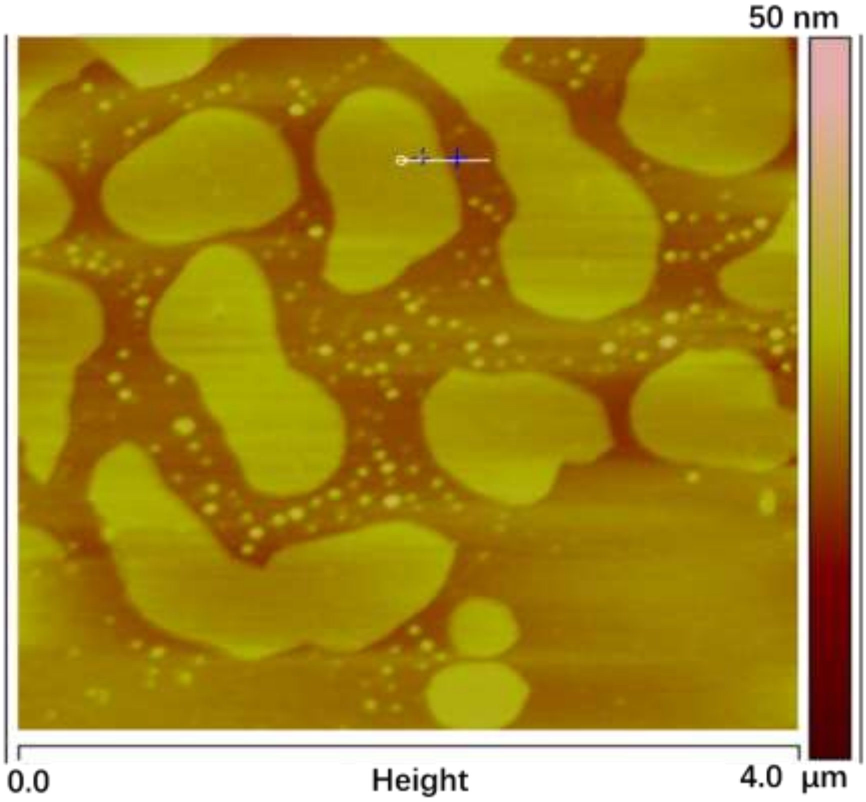

Fig. 17S The AFM of 2D CNS.

Table. 1S The Raman information of the as-derived samples.

	I_D/I_G	Peak1	Peak2	Peak3	Peka4	A_{sp3}/ A_{sp2}
1D CNF	0.84	1.939e+05	4.22e+05	4.677e+05	1.505e+05	1.15
2D CNS	0.94	1.337e+05	4.899e+05	3.632e+05	1.098e+05	0.83
3D CFW	0.92	4.084e+04	5.575e+05	2.749e+05	7.527e+04	0.58

Table. 2S The value of D_{Na+} for multi-dimension carbon structure at different cycles.

cycles		1st	5th	10th	20th	35th	50th	70th	100th	150th	200th
D_{Na+}	1D CFW	1.12	1.87	2.48	2.80	2.92	3.05	2.80	2.82	2.71	2.43
	2D CNS	0.10	0.19	0.24	0.28	0.25	0.43	0.34	0.32	0.50	0.36
	3D CFW	1.07	0.27	0.38	0.39	0.41	0.52	0.60	0.91	1.10	0.43

(D_{Na+}, $\times 10^{10}$ cm^2 s^{-1})
